# Overcoming Limited Access to Virus Infection Rapid Testing: Development of a Lateral Flow Test for SARS-CoV-2 with Locally Available Resources

**DOI:** 10.3390/bios14090416

**Published:** 2024-08-27

**Authors:** Estefanía S. Peri Ibáñez, Agostina Mazzeo, Carolina Silva, Maria Juliana Juncos, Guadalupe S. Costa Navarro, Horacio M. Pallarés, Virginia J. Wolos, Gabriel L. Fiszman, Silvia L. Mundo, Julio J. Caramelo, Marcelo J. Yanovsky, Matías Fingermann, Alejandro A. Castello, Andrea V. Gamarnik, Ana S. Peinetti, Daiana A. Capdevila

**Affiliations:** 1 Laboratorio de Inmunología y Virología (LIV), Departamento de Ciencia y Tecnología, Universidad Nacional de Quilmes, B1876BXD Buenos Aires, Argentinaacastello@unq.edu.ar (A.A.C.); 2 Instituto de Investigaciones Bioquímicas de Buenos Aires (IIBBA), CONICET, Fundación Instituto Leloir, C1405BWE Ciudad Autónoma de Buenos Aires, Argentina; amazzeo@qi.fcen.uba.ar (A.M.); csilva@leloir.org.ar (C.S.); mjuncos@leloir.org.ar (M.J.J.); gcosta@leloir.org.ar (G.S.C.N.); hpallares@leloir.org.ar (H.M.P.); jcaramelo@leloir.org.ar (J.J.C.); myanovsky@leloir.org.ar (M.J.Y.); agamarnik@leloir.org.ar (A.V.G.); 3 INQUIMAE (CONICET), Departamento de Química Inorgánica, Analítica y Química Física, Facultad de Ciencias Exactas y Naturales, Universidad de Buenos Aires (UBA), C1428EGA Ciudad Autónoma de Buenos Aires, Argentina; 4 Universidad de Buenos Aires (UBA), Instituto de Oncología Ángel H. Roffo, Área Investigación, C1417DTB Ciudad Autónoma de Buenos Aires, Argentina; jwolos@institutoroffo.uba.ar (V.J.W.); gfiszman@institutoroffo.uba.ar (G.L.F.); 5 Cátedra de Inmunología, Facultad de Ciencias Veterinarias, Universidad de Buenos Aires (UBA), C1427CWN Ciudad Autónoma de Buenos Aires, Argentina; smundo@fvet.uba.ar; 6 Instituto Nacional de Producción de Biológicos (INPB), ANLIS “Dr. Carlos G. Malbrán”, C1282AFF Ciudad Autónoma de Buenos Aires, Argentina; mfingermann@anlis.gob.ar; 7 Centro de Medicina Traslacional, Hospital El Cruce Néstor C., Kirchner, B1888 Buenos Aires, Argentina; 8 Instituto de Ciencias de la Salud, Universidad Nacional Arturo Jauretche, B1888 Buenos Aires, Argentina

**Keywords:** COVID-19, SARS-CoV-2, diagnostics, lateral flow tests, local development

## Abstract

The COVID-19 pandemic highlighted testing inequities in developing countries. Lack of lateral flow test (LFT) manufacturing capacity was a major COVID-19 response bottleneck in low- and middle-income regions. Here we report the development of an open-access LFT for SARS-CoV-2 detection comparable to commercial tests that requires only locally available supplies. The main critical resource is a locally developed horse polyclonal antibody (pAb) whose sensitivity and selectivity are greatly enhanced by affinity purification. We demonstrate that these Abs can perform similarly to commercial monoclonal antibodies (mAbs), as well as mAbs and other pAbs developed against the same antigen. We report a workflow for test optimization using nasopharyngeal swabs collected for RT-qPCR, spiked with the inactivated virus to determine analytical performance characteristics as the limit of detection, among others. Our final prototype showed a performance similar to available tests (sensitivity of 83.3% compared to RT-qPCR, and 90.9% compared to commercial antigen tests). Finally, we discuss the possibility and the challenges of utilizing affinity-purified pAbs as an alternative for the local development of antigen tests in an outbreak context and as a tool to address inequalities in access to rapid tests.

## 1. Introduction

Lateral flow tests (LFTs) were adopted on an unprecedented scale during the COVID-19 pandemic, demonstrating their feasibility and acceptability on a global basis. Professional use and self-tests have enabled LFT-based testing to be expanded beyond healthcare facilities and into homes and community settings such as workplaces, educational establishments, and large gatherings. However, this increase accentuated the challenges faced by low- and middle-income countries (LMICs), which struggled with unequal access to testing. The strong demand in high-income countries along with the short supply significantly impacted the availability in LMICs (a problem also seen for COVID-19 vaccines), despite the importance of LFTs in settings with limited molecular testing capacity and rural populations.

Remarkably, only 0.4% of the 3 billion tests conducted globally by 2022 were used in low-income regions, despite these regions representing 7.8% of the global population [1]. In response to these disparities, there is a critical necessity to build domestic manufacturing capacities within third-world countries to ensure a robust supply chain of LFTs for current and future pathogen outbreaks.

RT-qPCR tests can be rapidly developed for a new pathogen based on shared sequence data. By contrast, antigen LFT development requires weeks to months in the best of circumstances, including the design of capture receptors against target analytes. While various manufacturers have produced commercially available SARS-CoV-2 antigen LFTs during the pandemic (the first available LFT was in May 2020 [2]), access to validated tests remained difficult or cost prohibitive in LMICs. The World Health Organization (WHO) highlights the need for local suppliers and technical support, and a few reports have provided open-access architecture that facilitates the manufacture of SARS-CoV-2 LFTs using all commercially available reagents and materials [3,4]. However, in the context of the pandemic, *commercially* available is different from *locally* available, and many of the supplies were hard to obtain due to accessibility or cost issues. Thus, there is an urgent need to build domestic manufacturing capacity in every country.

One of the main bottlenecks in developing reliable LFTs involves the design of high-performance capture receptors (typically antibodies (Abs)) against the antigen. Obtaining high affinity and specific monoclonal antibodies (mAbs) could take several months to years in LMICs—a timeframe not conducive to rapid outbreak response. In contrast, while LMICs may not afford advanced and swift development for mAbs, the potential exists to leverage their already existing capabilities and experience in producing polyclonal Abs (pAbs) as active pharmaceutical ingredients for immunotherapeutic drugs, namely antivenoms [5,6,7]. Due to the lack of profit of antivenoms when compared to modern biopharmaceuticals, most large multinational pharmaceutical companies lost interest in these products. Local authorities in many LMICs thus had to strengthen their already existing traditional antivenom-producing facilities to meet their antivenom demands. As the majority of antivenom producers in Latin America are not-for-profit, public-sector manufacturers that meet domestic or sub-regional needs [8,9,10], they might also be able to provide pAbs for diagnostic purposes.

The percentage of immunoglobulins specific to the target antigen in the serum does not exceed 2–3% of the total immunoglobulins, and these specific pAbs are heterogeneous in their properties [11]. Nevertheless, by using different purification strategies, high-performance Abs can be separated and used in rapid antigen assays, offering sufficient clinical sensitivity to detect infectious patients in decentralized settings [11,12,13].

Another main bottleneck in developing LFT is the access to clinical samples. Ensuring the accuracy and reliability of these tests requires rigorous optimization and validation, which heavily relies on the availability of appropriate clinical samples. Some key points to consider regarding this bottleneck involve navigating ethical considerations and regulatory requirements; accessibility to facilities that are necessary for handling these samples, for instance, Biosafety Level 3 (BSL3) facilities; and ensuring the quality and standardization of clinical samples. Strategies to mitigate this bottleneck include establishing robust partnerships and exploring alternative sample sources where feasible.

In this context, part of our group has previous experience developing a commercial ELISA test for the serological detection of Abs against SARS-CoV-2 spike (S) protein [14]. The COVIDAR serologic test was generated early after the first COVID-19 case in Argentina, and over half a million tests have already been produced and distributed for free in the country. Our next goal was to create an LFT to address the increased demand for testing in Argentina following the end of the lockdown, which resulted in a slow increase in cases related to the opening of massive social activities [15]. We aimed to provide an effective solution to diagnose those affected.

Both the S and nucleocapsid (N) proteins have been candidates as detectable antigens in the development of rapid diagnostic methods [4,12,16,17,18]. The S protein is highly variable and easily mutates to escape population immunity [19,20], and genomic evidence has been found regarding the introduction and local transmission in Argentina of different variants in different geographical regions of the country. For instance, Gamma, Lambda, and Delta; and to a lesser extent, Alpha, Epsilon, and Zeta; the sporadic detection of Mu; and the circulation of mutations of interest (such as L452R and E484K in S) have been detected [15]. In contrast, N is a highly abundant protein expressed at the early stage of viral infection that can be directly detected in biological fluids and is also the most sensitive target for rapid Coronavirus diagnosis in general [21,22]. Moreover, the N protein is highly conserved among all SARS-CoV-2 variants [22,23], and even though it still undergoes slow evolutionary changes, N-binding Abs are highly cross-reactive, and the most immunogenic epitopes within this protein are not under selective pressure [22]. For the mentioned reasons, we aimed to develop an LFT prototype for SARS-CoV-2 detection in respiratory secretions based on the N protein as the main antigenic target.

## 2. Materials and Methods

### 2.1. Equipment, Reagents, and Materials

The CM5000™ guillotine cutting module, LM5000™ manual lamination system, and ZX1010™ benchtop dispenser workstation were purchased from BioDot Inc. (Irvine, CA, USA). The lateral flow reader iPeak^®^+ was purchased from IUL Instruments (Barcelona, Spain). The sample (CFSP238000), conjugate (GFDX203000), and wicking pads (CFSP223000) were purchased from Merck. Nitrocellulose membranes CN95 and HFC (7502, 9002, and 13502) were purchased from Sartorius (Goettingen, Germany) and Millipore^®^ (Merck KGaA, Darmstadt, Germany), respectively. Finally, 6 cm wide backing cards were purchased from Diagnostic Consulting Network Inc. (Carlsbad, CA, USA).

Bovine Serum Albumin (BSA), protein A, and all reagents to prepare the saline and buffer solutions were purchased from Merck KGaA (Darmstadt, Germany), unless otherwise stated, and used as received. Concentrated nitric and hydrochloric acids were purchased from Anedra (Research-AG, Buenos Aires, Argentina) and used as received. Gold (III) chloride was purchased from Sigma-Aldrich^®^ (Merck KGaA, Darmstadt, Germany) and used as received.

The aptamer used for N protein recognition (TTTTTGCTGGATGTCGCTTACGACAATATTCCTTAGGGGCACCGCTACATTGACACATCCAGC) was obtained from Integrated DNA Technologies (Coralville, IA, USA) with a thiol modification at the 5′ end and a biotin modification at the 3′ end [24]. The aptamers were used without further purification.

Rabbit anti-horse IgG (whole molecule) peroxidase antibodies were purchased from Sigma-Aldrich^®^ (Merck KGaA, Darmstadt, Germany). A goat anti-llama IgG (H + L) secondary antibody, HRP, was purchased from Thermo Fisher Scientific (Waltham, MA USA). The goat anti-mouse IgG HRP-conjugated antibody was purchased from R&D Systems, Inc. (Minneapolis, MN, USA). Commercial rabbit anti-N mAbs were purchased from Creative Diagnostics (Shirley, NY, USA). All commercial antibodies were used as received. Buffer exchanges were typically performed using 10 K and 30 K 500 μL Amicon^®^ Ultra Centrifugal (Merck KGaA, Darmstadt, Germany) filters. Protein concentration was routinely determined by absorbance measurements at 280 nm using a Thermo Fisher Scientific (Waltham, MA USA) NanoDrop 2000 spectrophotometer, using deionized water as blank.

### 2.2. SARS-CoV-2 Antigens Production and Purification

#### 2.2.1. Expression and Purification of SARS-CoV-2 Nucleocapsid Protein N-Terminal Domain

DNA sequence coding for the N-terminal domain of the SARS-COV2 N protein was cloned in the bacterial expression vector pET-22b(+) (Merck KGaA, Darmstadt, Germany, Novagen cat. 69744) using the restriction sites BamHI and SacI. This vector adds a 6xHis tag at the C-terminal end. Insert identity was verified by DNA sequencing of both strands. *E. coli* BL26 cells were transformed and cultured at 37 °C in 100 mL of Luria-Bertani (LB) medium with 100 μg/mL ampicillin. Next, a 1:20 dilution in 1 L of LB was incubated for 1 h at 37 °C and then for 30 min at 25 °C. Transcription was induced with 0.5 mM IPTG for 16 h at 18 °C. Bacteria were harvested by centrifugation at 1000 g for 12 min and resuspended in 100 mL of 50 mM Tris-HCl, 150 mM NaCl, 10 mM imidazole, and 1 mM PMSF at pH 7.5. Lysozyme (0.1 mg/mL) and Triton X-100 (1% *v*/*v*) were added, and the mixture was incubated at 25 °C for 45 min. After sonication for 3 min, samples were clarified by centrifugation for 30 min at 15,000× *g*. Proteins were purified by immobilized metal affinity chromatography (IMAC) using Ni^2+^ and eluted with 0.5 M imidazole. Fractions were analyzed using SDS-PAGE 15:3, and those with the highest concentration of N NTD were pooled and stored at -80°C with 10% glycerol. Next, proteins were purified by size exclusion chromatography with a Superdex-200 HR10/30 Column (Cytiva^TM^, Marlborough, MA, USA) in phosphate-buffered saline (PBS).

#### 2.2.2. Expression and Purification of SARS-CoV-2 Spike Protein (S)

Expression and purification of the recombinant RBD domain of the Spike protein was performed as previously reported. Briefly, a recombinant version of the RBD protein (rRBD) was created after transfection in FreeStyle 293-F (293-F) cells, as described by Ojeda et al. [19].

#### 2.2.3. SARS-CoV-2 Pseudotyped Vesicular Stomatitis Virus (VSV)

Viral stocks were obtained as described by Gonzalez Lopez Ledesma et al. [25]. These stocks were titrated by fluorescence-forming units per ml (FFU/mL) in Vero cells.

#### 2.2.4. Inactivated SARS-CoV-2 Particles

SARS-CoV-2 particles were cultivated in Vero cells and chemically inactivated [26,27,28]. Quantification was performed by RT-qPCR in the COVID-19 unit of the Universidad Nacional de Quilmes (UNQ) [29] using Quantabio Script One-Step RT-PCR (Genbiotech SRL, Autónoma de Buenos Aires, Argentina) for RNase P detection and SARS-CoV-2 Nucleic Acid Detection Kit (Multiplex Real Time RT-PCR) (TransGen Biotech Co., Ltd., Beijing, China). The samples were analyzed according to the manufacturers’ recommendations, and the stock concentration was determined to have a Ct = 23.

### 2.3. Anti-N and Anti-S Recognition Elements Production and Purification

#### 2.3.1. Anti-N Mouse mAbs

The production of anti-N mouse mAbs was carried out using the hybridoma technology. Briefly, we immunized Balb/c mice i.p. every 15 days with 50 µg/mouse of purified N protein for 60 days. The first inoculations were with the addition of incomplete Freund’s adjuvant, and the last booster was inserted intravenously. The mouse with the highest serum titer was selected by the ELISA test (already described). Splenocytes were extracted and fused with the NS0 myeloma cell line (ATCC # CRL-1851) using 45% PEG 1500. The hybridomas were selected by growth in the HAT medium (complete medium: RPMI-1640 medium supplemented with 10% fetal bovine serum, containing hypoxanthine, aminopterin, and thymidine). The hybrid clones secreting the specific mAbs were individualized by successive ELISA screenings and then cloned by limiting dilution. Two of these hybridoma clones were established by in vitro continuous growth and isolated for their high-affinity mAbs production.

Mouse ascitic fluid was diluted 100-fold and purified by affinity chromatography using three 5 mL HiTrap^®^ Protein G columns purchased from Cytiva^TM^ connected in series to an ÄKTA FPLC purification system. The fractions were eluted in a 0.1 M pH 2.8 glycine buffer and collected in a 10% 1 M pH 8 Tris buffer for quick neutralization.

#### 2.3.2. Hyperimmune Horses’ Plasma Production

Horses’ pAbs against N and S antigens were generated essentially as described by González Viacava et al. [30]. Briefly, 4 mixed-breed 4–10 years-old, 300–450 kg horses (2 horses per antigen) were submitted to two consecutive hyperimmunization schedules. During the first cycle (I), horses were initially primed subcutaneously with 0.5 mg antigen in 30% (*v*/*v*) complete Freund adjuvant (CFA) in saline, boosted two weeks later with 1.0 mg antigen in 30% (*v*/*v*) incomplete Freund adjuvant (IFA) *v*/*v* in saline, and boosted 3–4 times at weekly intervals with 1.5 mg antigen in a 20% (*v*/*v*) dilution in saline of a stock Al(OH)_3_ suspension. After a 60–120 days resting period, a second immunization cycle (R cycle) was conducted. Horses were initially boosted subcutaneously with 0.5 mg antigen in 30% (*v*/*v*) IFA *v*/*v* in saline, and 3–4 sequential 1.5 mg antigen doses using Al(OH)_3_ suspension as adjuvant were administered subcutaneously at weekly intervals. Seven days after the end of the R cycle, hyperimmunized horses’ blood was collected on two sequential days, and their plasma was separated after citrate addition and conserved refrigerated until use.

#### 2.3.3. Llama Sera Production

Llama pAbs against N were generated essentially as described by De Simone et al. [31]. Briefly, two llamas were immunized with the antigen. First, 0.5 mg antigen in saline was emulsified with an equal volume of IFA (Sigma Chemicals Co., Saint Louis, MO, USA) and injected i.m. in the bicep femoral muscle (day 1), repeating the process on day 14 with 1.0 mg antigen in IFA and boosted 2 times at 28 and 35 days with 1.5 mg antigen in IFA. Llamas were bled by the jugular vein on day 50. Sera were separated and conserved refrigerated until use.

#### 2.3.4. Anti-N Llama and Horse pAbs Purification

Horse plasma and llama sera were purified in a two-step procedure. An initial caprylic acid precipitation was carried out for non-immunoglobulin content reduction, essentially as described by Nudel et al. [32]. The supernatant was further purified by elution in a SARS-CoV-2 N protein affinity column. The fractions were eluted in a 0.1 M pH 2.8 glycine buffer and collected in a 10% 1 M pH 8 Tris buffer for quick neutralization. The fractions were analyzed by SDS-PAGE to determine the level of contamination with other plasma proteins.

### 2.4. ELISA Assays

**Figure 1:**
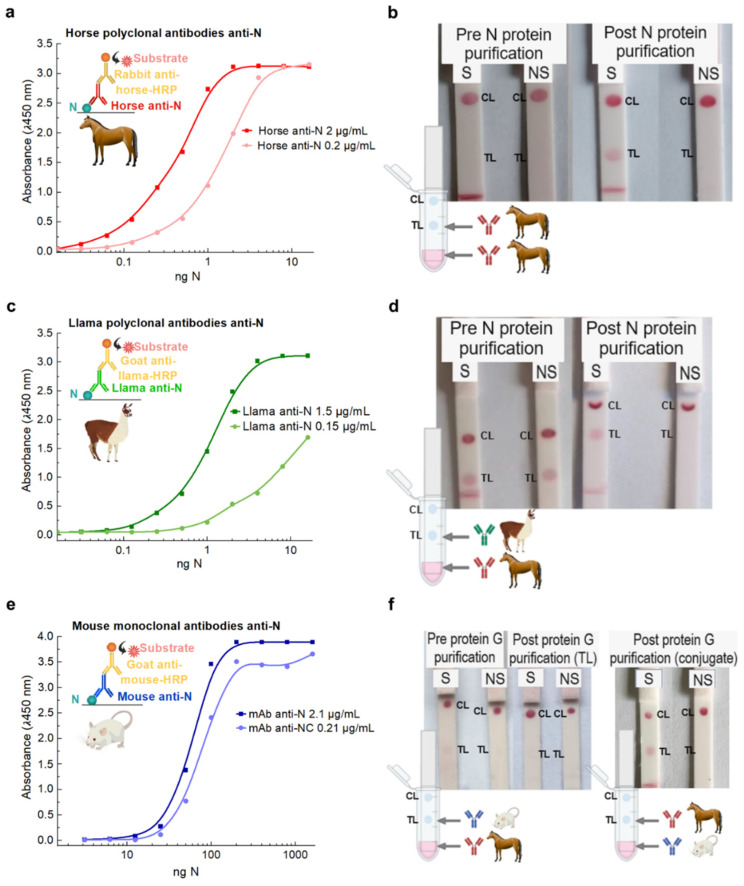
(**a**) ELISA assay results curves for detection of recombinant N protein with 2 and 0.2 µg/mL horse anti-N pAbs showing the recognition of the N protein. (**b**) Half-strip assays testing sample buffer with (Spiked, S) and without (Non-spiked, NS) 10 ng N protein addition, showing the performance of 2 µg/strip of horse anti-N pAbs as the capture element in the test line (TL) and Protein A in the control line (CL) before and after N protein column affinity purification with AuNPs-horse anti-N conjugate. (**c**) ELISA assay results curves for detection of recombinant N protein with 1.5 and 0.15 µg/mL llama anti-N pAbs showing the recognition of the N protein. (**d**) Half-strip assays testing sample buffer with (Spiked, S) and without (Non-spiked, NS) 10 ng N protein addition showing the performance of 1.5 µg/strip llama anti-N Abs as the capture element in the test line (TL) and Protein A in the control line (CL) before and after N protein column affinity purification with AuNPs-horse anti-N conjugate. (**e**) ELISA assay results curves for detection of recombinant N protein with 2.1 and 0.21 µg/mL mouse anti-N mAbs showing the recognition of the N protein. (**f**) Half-strip assays testing sample buffer with (Spiked, S) and without (Non-spiked, NS) 10 ng N protein addition showing the performance of 2.1 µg/strip mouse anti-N mAbs as the capture element in the test line (TL) and Protein A in the control line (CL) before and after Protein G column affinity purification with AuNPs-horse anti-N conjugate (left) compared to the same Ab as the detection element in the AuNPs-Ab conjugate using strips with 1 µg/strip horse anti-N Ab as the capture element (right).

ELISA assays were performed to determine the recognition capacity of the purified Abs by the N and S proteins. Washing solution, sample diluent, chromogen, and substrate were used from the COVIDAR ELISA kit [14]. Briefly, ELISA microtiter plates (Jet Bio-Filtration Co., Ltd., Guangzhou, China) were coated overnight at 4 °C with different concentrations of antigen protein and BSA (as a control antigen) diluted in PBS. The plates were afterwards washed six times with the COVIDAR washing solution. Then, 100 µL of each Ab solution was diluted in the COVIDAR sample diluent to different final concentrations and was added to the antigen-coated wells; the plates were then incubated at 37 °C for 1 h and washed. Peroxidase-conjugated secondary Abs (rabbit anti-horse IgG 1:1000, goat anti-llama IgG 1:5000, and goat anti-mouse IgG 1:100) diluted in the COVIDAR sample buffer was added at 100 µL/well, incubated for 30 min at 37 °C, and washed. Bound Abs and conjugates were then revealed with chromogen and substrate from the COVIDAR ELISA kit. The optical density (OD) was measured at 450 nm (OD450). The control antigen OD was subtracted from each measurement to obtain the results (as shown in Figure 1).

### 2.5. Gold Nanoparticles (AuNPs)—Ab Conjugation Process

First, 20 nm AuNPs were synthesized from HAuCl_4_ and sodium citrate following a modified Turkevich protocol [33]. Briefly, the needed glassware was repeatedly washed with *aqua regia* and then thoroughly rinsed with ultrapure water. The HAuCl_4_ 0.01% solution was brought to boil under constant stirring. At this point, the fresh 1% sodium citrate solution was quickly added. The suspension was kept at a boiling temperature under constant stirring for 10 min, then placed in a cool bath until room temperature was reached. These particles were used as seeds and further grown using a similar procedure. A diluted solution of AuNP seeds was mixed with the fresh 1% sodium citrate solution and brought to a boil under constant stirring. Once the boiling point was achieved, HAuCl_4_ 0.2% was quickly added. This step was repeated twice, at 5 min and 7 min after the first addition. The suspension was kept at a boiling temperature under constant stirring during the process. Once the suspension turned dark red, it was placed in a cool bath until it reached room temperature. The suspension was stored at 4 °C and kept in the dark until use. Both seeds and full-grown AuNPs were characterized by UV–vis measurements and scanning electron microscopy experiments performed using a Thermo Fisher Scientific Quanta 250 microscope (Instituto Nacional de Tecnología Industrial (INTI), Buenos Aires, Argentina). The resulting images were analyzed using ImageJ software 1.54j (Figure S1).

To optimize conjugation variables, such as Ab concentration and conjugation pH, the stability of different aliquots towards the addition of NaCl was studied. Firstly, the Abs were buffer exchanged to a low ionic force media, such as a 12 mM inorganic phosphate buffer (Pi), and solutions with Ab concentrations of 40, 80, and 160 ng/μL were prepared. Aliquots of the AuNP suspensions were brought to a determined pH value between 4 and 9 with 0.2 M K_2_CO_3_ and incubated with 10% *v*/*v* of each Ab solution for 10 min at room temperature. Afterwards, 20% *v*/*v* of NaCl 10% was added to each aliquot and incubated for 30 min at room temperature. Color changes and precipitate formation were monitored once the incubation was over. The aliquots for which there were no noticeable changes were chosen as appropriate conditions for conjugation. Additional UV–vis measurements were performed as a quality control. Final conjugation conditions were further optimized based on performance when more than one condition was suitable according to the NaCl stability test.

Horse pAbs, mouse mAbs, and commercial rabbit mAbs were physically adsorbed by mixing AuNPs (OD between 1 and 1.2) and Ab solutions in 12 mM inorganic phosphate buffer (Pi) in an approximate 100:1 volume ratio and incubated at room temperature for 10 min in the dark. Optimal pH and Ab concentration conditions were chosen for each Ab based on the optimization experiments previously described. Conjugation parameters for each Ab type can be found in Table S1. The particles were further blocked by incubation with varying volumes of BSA 10% and PEG-20000 1% unless indicated otherwise. The resulting mixture was centrifuged at 8000 RPM for 15 min, and the obtained pellet was resuspended in an optimized buffer adjusted to pH 7.5 and a final OD of 10 unless indicated otherwise. Absorption spectra of the obtained AuNPs-Ab conjugates were measured as a quality control routine, in which λ_MAX_ was expected to be around 530 nm (Figure S1).

### 2.6. LFA Assay Preparation

Firstly, the nitrocellulose membranes were assembled on a 6 × 30 cm backing card. Typically, 0.25 µg/µL protein A in PBS buffer was used for the control line (CL) and 1–2 µg/µL anti-N Abs in PBS was used for the test line (TL). Both reagents were dispensed on the nitrocellulose membranes using the BioDot BioJet Quanti dispensers. The dispensing rate was 3 µL/cm for the TL and 2 µL/cm for the CL. Both lines were located approximately 7 mm apart. Once the reagents were dispensed, the membranes were allowed to dry for 1 h at 37 °C. The membranes were afterward blocked using a PBS buffer containing BSA and 3% sucrose, then dried likewise.

In parallel, the conjugate suspension was dispensed on the glass fiber conjugate pad at 60 µL/cm using the AirJet feature in the BioDot benchtop dispenser. The pads were afterwards dried for 1 h at 37 °C.

The sample pad was pretreated with the sample buffer and dried at 37 °C for 1 h. The wicking and conjugate pads were cut and used without any pretreatment.

All elements were stored in the dark at room temperature in low-humidity conditions until assembly. The pads and the membrane were overlapped when assembled to ensure a continuous flow through the strip, as shown in Figure S2. Finally, the resulting pieces were cut into 3.7 mm wide strips to fit commercial LFT cassettes bought from Zhuhai Ideal Plastic Product Co., Ltd. (Zhuhai City, China).

#### Half-Strip Assays

Half-strip experiments were performed with test strips devoid of sample and conjugate pads. Typically, protein A (0.25 µg/µL in PBS) was dispensed using a micropipette as a 0.5 µL dot below the wicking pad, and capture Ab solutions of different concentrations were dispensed as 0.5–1 µL dots 0.7 cm below the control dot. The half-strips were tested by dipping into a mixture of sample buffer and conjugate suspension, with or without the addition of N protein or inactivated SARS-CoV-2 particles.

### 2.7. LFT Evaluation and Sample Testing

To ensure that the assembled tests, including the sample and conjugate pads, were working properly, each strip was placed inside a plastic cassette in a low-humidity room. To evaluate their performance, we tested 100 µL of the sample buffer (typically, PBS with 1% Triton x-100) with and without the addition of the N protein or inactivated SARS-CoV-2 particles, dispensed dropwise at the sample well. The results were read after 20 min. The optimized parameters are outlined in Table 1.

**Figure 2:**
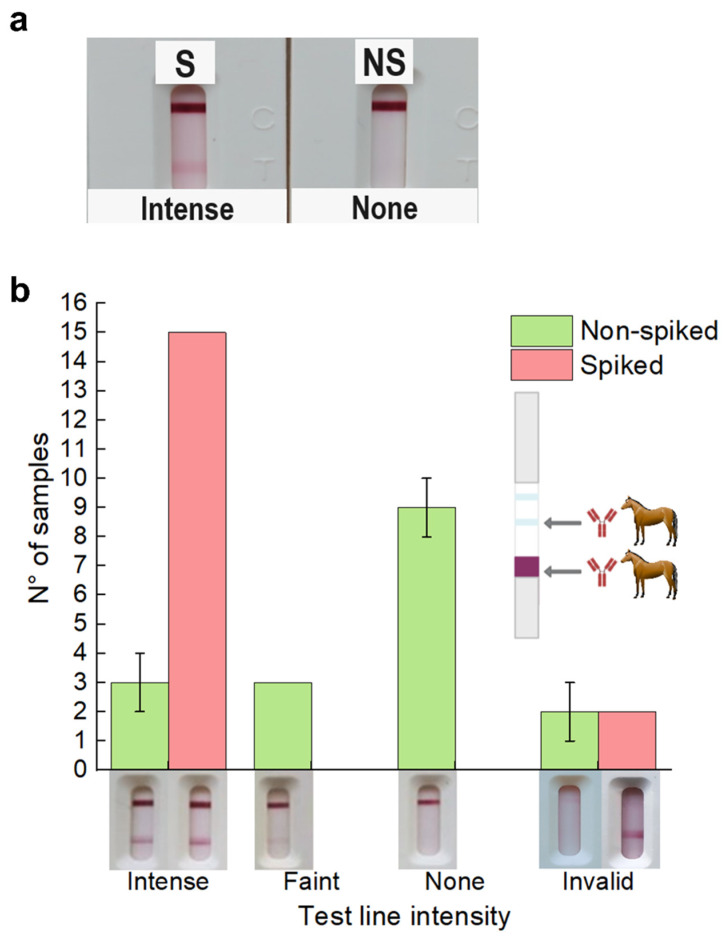
(**a**) Full strip assays using sample buffer with (S) and without (NS) inactivated virus (1 and 2) on buffer. Buffer tests showed an intense test line signal for the spiked sample and no signal for the non-spiked sample. (**b**) The histogram showing the relative intensity (qualitatively) for the tested RT-qPCR negative nasopharyngeal samples. Examples of the possible results obtained with full strip assays on RT-qPCR negative nasopharyngeal swabs are shown below the histogram for reference. Although most of the spiked samples showed a very intense test line signal, a high proportion of non-spiked samples showed some sort of non-specific coloration. Moreover, a proportion of spiked and non-spiked tests gave invalid results given the absence of a control line. In all cases, the strips’ test lines were dispensed with 1 µg horse anti-N pAbs with PBS as the application buffer per strip on a Sartorius UniSart CN95 membrane. The signal intensity on the TL was qualitatively measured by the naked eye by three independent users. The error bars were then estimated by the SD of these three independent measurements.

For most of the experiments, the signal intensity on the TL was qualitatively measured by the naked eye by three independent users using the scale shown in Figure 2 as a reference. The error bars were then estimated by the SD of these three independents measurements. For LOD and volunteers’ nasal self-extracted experiments, test results were read using an iPeak^®^+ lateral flow reader.

The LOD was calculated using a low-concentration dilution [34], as follows: LoD = LoB + 1.645(SD low concentration sample), where LoB = mean blank + 1.645(SD blank).

RT-qPCR assays of negative SARS-CoV-2 nasopharyngeal samples were performed at the INBIRS Institute.

Nasopharyngeal samples positive by RT-qPCR for SARS-CoV-2 with Ct values ranging from 15 to 35 (positivity criteria: Cts for ORF1a and N genes < 36 or Ct for N gene < 36) were used at INBIRS for the evaluation of the prototype and compared with SARS-CoV-2 MonlabTest^®^ (Lot. A1087SCV695049) to estimate sensitivity.

Nasal samples were self-extracted by volunteers from Fundación Instituto Leloir and Universidad Nacional de Quilmes. Typically, two nasal swabs were used to collect samples from each nostril separately. One of the samples was extracted in 400 µL of the sample buffer, of which 100 µL (three drops) were pipetted and used to run the tested prototype. The other sample was extracted in 400 µL of the commercial sample buffer and used to run the commercial tests according to the manufacturer’s recommendations, to make a relevant comparison. The commercial tests evaluated were the Abbott PANBIO™ COVID-19-Ag rapid test (Lot. 41ADF059A) and SARS-CoV-2 MonlabTest^®^ (Lot. A1087SCV695049).

### 2.8. Conjugate Pad Transplantation

Conjugate pad transplantation [35] was carried out between the prototype (described in Table 1) and the Artron COVID-19 Rapid Antigen Test Kit (Lot. 210816). Briefly, the Artron cassette was disassembled, and the conjugate pad was carefully removed and replaced with the prototype test conjugate pad, as described in Table 1. Simultaneously, the Artron test conjugate pad was relocated between the sample pad and the nitrocellulose membrane of the test presented here. Lastly, both cassettes were reassembled and tested with their respective running conditions.

## 3. Results and Discussion

### 3.1. Locally Developed Anti-N Abs Performance Comparison

We first thought to test and compare the different developed Abs as well as the impact of the purification protocol on the analytical performance. The comparison was performed by ELISA assays and half-strip tests (Figure 1 and Figure S3). We decided to take advantage of the earlier development of anti-SARS-CoV-2 sera to obtain anti-RBD horse pAbs as a therapeutic tool against COVID-19 [30] in order to perform a comparison between anti-N and anti-S horse pAbs. While the sensitivity of horse anti-S pAbs reported in ELISA is remarkable for both the protein and the VSV particles with the S protein [14], the limit of detection (LOD) for viral particles is not compatible for their use for traditional rapid diagnostics [36] (Figure S3). On the other side, all anti-N pAbs, as well as the mouse mAb, showed an LOD for the N protein in ELISA assays suitable for rapid testing of nasopharyngeal swab samples [36,37,38] (Figure 1a,c,e and Figure S3). The ELISA results for pAbs detected concentrations of N protein in the same order of magnitude as those reported in the supplier specifications for typical commercial mAbs (1–10 ng/mL); this suggests that they are comparable in terms of sensitivity [39,40,41]. As an alternative that is easy to produce locally and that was readily available for N protein detection, we evaluated the performance of anti-N aptamers (Figure S3) [24]. Unfortunately, even though we could attain the same performance as reported in the original publication using the N protein in the buffer, the LOD was not comparable with pAbs. It is important to highlight that functional DNA has several advantages when it comes to overcoming limited access to virus infection rapid testing with locally available resources, as the sequences can be found in published articles, and the synthesis of oligonucleotides is available worldwide and locally produced in most countries [42,43,44,45]. Moreover, while we focused on the first available aptamer specific to N protein detection, better results may be achieved if improved alternatives are used [45,46,47].

We tested the selected Abs in the context of an LFT by performing half-strip assays before and after different purification steps using a sample buffer with and without the addition of the N protein, labelled S and NS, respectively (Figure 1b,d,f). These assays are valuable for assessing the correct functioning of the recognition elements and other materials during LFT optimization. Thus, they were used throughout this study as quality control checkpoints for the performance of different LFT elements. Here, as the goal was to compare different Abs, we performed all the assays with standard conditions, which showed a robust performance (namely, Sartorius UniSart CN95 membranes with 0.15% BSA blocking and PBS containing 1% Triton x-100 as a sample buffer).

Interestingly, when dispensed in the test line, llama and horse anti-N pAbs and mouse anti-N mAbs yielded tests with very different performances. In the case of horse anti-N pAbs, the first purification step yielded pure IgG Abs, as caprylic acid precipitation can successfully isolate these proteins from the albumin-rich sera of the immunized animals (Figure S4a). However, when these IgG Abs were directly applied on the TL, no colorimetric signal was observed with the spiked sample, indicating a false negative result (Figure 1b). In contrast, when the same Abs were further purified by elution through the N protein affinity column, 5% of the proteins were tightly bound to the column and only eluted when a low pH glycine buffer was used (Figure S4a). Then, when these fractions were neutralized, buffer exchanged, and dispensed on the test line, a colorimetric signal was clearly seen for the spiked sample and absent in the non-spiked sample (Figure 1b). This shows that N protein affinity column purification is a key strategy for enabling the utilization of horse pAbs in rapid antigen testing, as it selectively concentrates on the less abundant, more specific anti-N pAbs present in the sera. Thus, we conclude that the purification step is likely selecting the antibodies with a higher affinity for the N protein.

On the other hand, when the half-strip assay was performed with caprylic-acid precipitated anti-N llama pAbs, it yielded a non-specific colorimetric signal in non-spiked samples, while N protein affinity-purified llama Abs showed a strong signal only when evaluating spiked samples (Figure 1d). This observation indicates that affinity purification also has a remarkable effect on the analytical performance of llama anti-N Abs, which seem to be even more significantly enriched in this purification step (Figure S4b). Interestingly, in this case, the purification protocol helped eliminate false positives as well as increasing the test’s sensitivity.

Finally, half-strip tests were also performed for mouse anti-N mAbs dispensed on the test line (Figure 1f). As expected from the ELISA, where the signal was obtained at comparatively higher concentrations (Figure 1e), a faint signal was seen when evaluating spiked samples with either pre- or post-protein G affinity-purified mAbs as capture elements (Figure S4c). We also note that pAbs can bind to different epitopes of the N protein, which likely improve their sensitivity when used as capture elements [3]. Altogether, mouse mAbs were not further used as capture reagents. Instead, when mouse mAbs were used as the detection element, a strong colorimetric signal was observed when evaluating spiked samples, indicating that mAbs are well suited for LFT sandwich assays (Figure 1f, right).

Given the superior analytical performance of pAbs as capture elements, the comparatively lower yield of affinity purification of llama pAbs, and the increased availability of horse pAbs, we focused on optimizing the application conditions in the test line for affinity-purified horse anti-N pAbs.

The different anti-N pAbs were then conjugated to AuNPs, and their performance was compared against commercial antibodies (rabbit anti-N mAbs, anti-SARS-CoV-2 NP, clone NJ1, Creative Diagnostics) (Figure S5). Conjugates were obtained at various Ab concentrations ranging from 40 to 320 ng/µL and pH values ranging between 4 and 9, specific to each antibody, as outlined in Table S1 and Figure S6. While stability and suitable pH ranges were generally improved at higher Ab concentrations, the colorimetric signal in the LFT decreased (Figure S6). Moreover, for horse pAbs, when the conjugation pH was set to 6, the conjugate pad remained notably colored after the test run was completed, indicating a low conjugate release rate, undesirable for LFT tests. Thus, the conjugation conditions were therefore defined jointly by the stability tests and the overall performance in LFT.

Optimal conjugation pH and concentration were 7 and 80 ng/µL, respectively, for horse pAbs; pH 6 and 160 ng/µL for mouse mAbs; and pH 6 and 160 ng/µL for commercial mAbs. The performance of the different conjugates was overall comparable when tested in a buffer-containing N protein (Figure S5a). The performance of horse anti-N pAb conjugates was better than mouse mAb and rabbit mAb conjugates considering both the intensity in full strips assays of spiked nasopharyngeal swabs and the percentage of false positives, as discussed below (Figure S5b). While comparable, the commercial rabbit mAb conjugate showed a decrease in signal intensity for spiked samples, which may be indicative of a slightly lower sensitivity under this condition. Therefore, we decided to continue optimizing a test using anti-N horse pAbs both as detection and capture elements.

### 3.2. Testing in Spiked and Non-Spiked Negative RT-qPCR Nasopharyngeal Swabs

We then turned to optimizing the sample buffer composition to enable direct detection of inactivated SARS-CoV-2 particles. In the solution, the recognition elements do not necessarily perform similarly when evaluating samples containing viral particles instead of free viral components (i.e., N protein in this case), as the antigen must be properly exposed first [48,49]. SARS-CoV-2 particles cultivated in Vero cells and chemically inactivated were readily available, easy to quantify, and served as a good model for spiking relevant negative samples [26,27,28].

We first compared the performance of two detergents to evaluate antigen detection efficiency. On the one hand, Tween 20 and Triton X-100 at a concentration of 0.5% did not interfere with N protein detection (Figure S7a). On the other hand, Triton X-100 allowed a broader dynamic range and performed better at high concentrations of viral particles (Ct = 24, Figure S7a). Triton X-100 optimal concentration was 1%, as higher concentrations resulted in non-specific binding (NSB) and a lower dynamic range (Figure S7b). Under these conditions, full strip assays performed well on the spiked (S) and non-spiked (NS) sample buffers (Figure 2.

Once we had a functioning test that accurately detected inactivated SARS-CoV-2 particles in a buffer solution, we started testing nasopharyngeal samples. One key complication when developing rapid diagnostic tests locally during an outbreak is the difficulty accessing a BSL3 facility and working with nasopharyngeal samples from patients with symptoms compatible with a SARS-CoV-2 infection. This heavily limits the ability of locally developing rapid diagnostic tools. To avoid these complications, we took advantage of the local availability of inactivated SARS-CoV-2 and worked with nasopharyngeal samples which had been previously tested by RT-qPCR for SARS-CoV-2 and came back negative. This was available through a collaboration with the institutes in charge of performing the RT-qPCR for diagnostics of patients with compatible symptoms and Ct > 36.

Each sample was evaluated with (S) and without (NS) the addition of inactivated viral particles to estimate the specificity of the full assay and the influence of biological fluids. While most spiked samples showed intense colored signals on the test line, many non-spiked samples also showed faint or intense coloration as a result of NSB, resulting in 6 out of 15 (40%) false positive results (Figure 2b). This NSB percentage was even higher when conjugates with mAbs were used instead of the horse pAbs (Figure S5b). These results highlight the need to evaluate the performance of relevant samples, which is not the case in most of the published work on developing new diagnostics tools, in which detection of antigens in a buffer solution is considered to be a sufficient proof of concept and selectivity is often not evaluated [17,50,51,52,53,54,55].

As a first attempt to reduce NSB, we explored different application buffers for dispensing horse pAbs in the test line (Figure S8a). We tested distinct buffers with a low ionic strength to favor electrostatic interactions, testing different pH values close to the isoelectric point of the capture Abs [3]. It is interesting to note that, while horse anti-N Abs dispensed in PBS yielded false positive results in experiments using nasopharyngeal swab samples, these did not produce a strong false positive signal in buffer samples. When dispensed in a buffer of pH 6.5 25 mM MES, the false positive rate on nasopharyngeal swab samples was significantly reduced (Figure S8b).

While changing the application buffer for the test line gave rise to a significant improvement, the percentage of false positive results observed was still above what is acceptable for LFTs [49]. The nasopharyngeal samples tested here allowed us to optimize our assay in more relevant conditions than the sample buffer or commercial saliva samples, accounting for the variability that arises from different patients. It should be noted that most of these samples are preserved in physiological solution or PBS, which can favor artifact generation and NSB. Nonetheless, we reasoned that preventing the NSB observed in these samples was key to obtaining an LFT that can be compared to commercial tests and can be locally produced. Thus, we turned to three distinct strategies to decrease NSB on these samples, while controlling that sensitivity remained acceptable using spiked samples.

### 3.3. Tackling Non-Specific Binding by Modifying Membrane, Sample Buffer, and Conjugate

**Figure 3:**
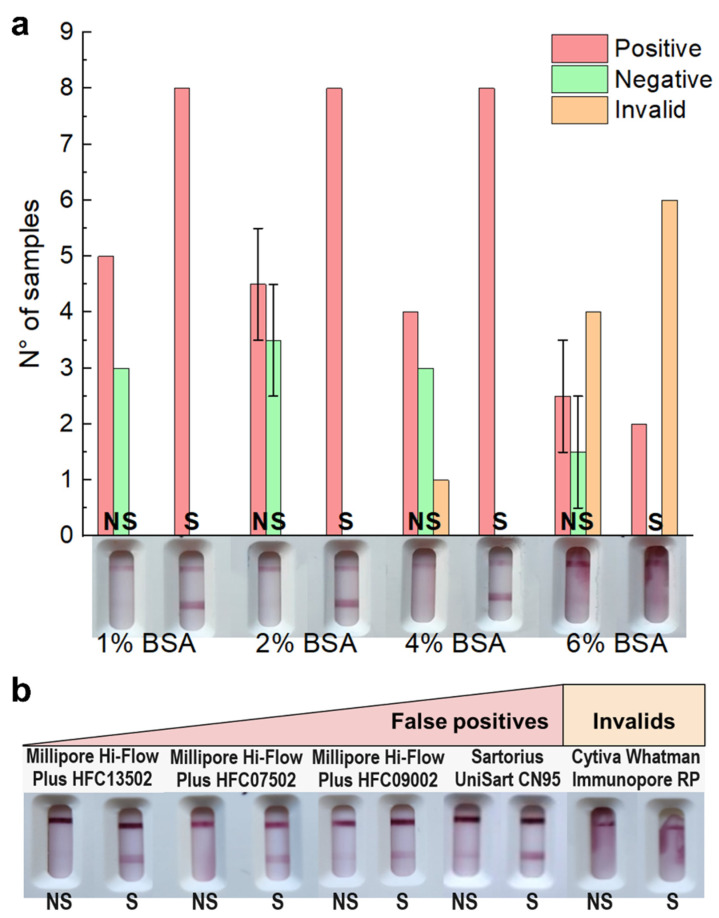
(**a**) Full strip assays using spiked (S, inactivated virus) and non-spiked (NS, sample buffer) RT-qPCR nasopharyngeal swab samples ran in UniSart CN95 membranes previously blocked with increasing amounts of BSA. In all cases, the strips’ test lines were dispensed with 1 µg horse anti-N per strip. The signal intensity on the TL was qualitatively measured by the naked eye by three independent users. The error bars were then estimated by SD of these three independents measurements. (**b**) Full strip assays using spiked (S, inactivated virus Ct = 26) and non-spiked (NS, sample buffer) RT-qPCR nasopharyngeal swab samples ran in five types of membranes with different flow rates. In all cases, the strips’ test lines were dispensed with 2 µg horse anti-N per strip with 2% BSA in the blocking buffer.

Typical sources of NSB are nonspecific interactions between the conjugated labels and the capture Abs and/or membrane as well as physical trapping of the conjugated labels in the porous membrane [48,49]. Thus, we re-evaluated the nitrocellulose membrane blocking (Figure 3a) [48,49,56]. Moreover, the shelf-life of LFT can be preserved by adding proteins and sugars that serve as stabilizing agents, protecting the tests from various damaging factors (e.g., temperature, free radicals, proteolytic activity, heavy metals) remaining in the environment [57]. Once the test and control line reagents were dispensed, the nitrocellulose membranes were coated with a blocking buffer (PBS buffer with 3% sucrose and BSA) to avoid NSB. The concentration of sucrose was determined based on our group’s previous experiences and literature [57]. We also tested two of the most common blocking agents used in LTF 1–2% *w*/*v* BSA and 1–2% *w*/*v* casein [3]. LFT containing casein in the blocking buffer resulted in a poor test flow, while BSA produced better results. Then, BSA concentration in the blocking buffer was evaluated in the range from 0.15% (original blocking buffer) to 6% in the spiked and non-spiked sample buffer and RT-qPCR negative nasopharyngeal swabs, and the results were compared to pin out the condition which minimized invalid and false positive results. BSA concentrations from 0.15% to 1% were tested with a different set of samples, and the results are shown in Figure S9. In a set of 30 samples, strips blocked with a 4% BSA or higher concentration resulted in a considerable rate of invalid tests that did not run properly, so these alternatives were discarded not based on NSB but on the effect on the test flow [58]. Although none of the blocking protocols eliminated the false positive results, these were minimized when using 1–2% BSA in the blocking buffer (Figure 3a).

To evaluate the possible physical trapping of the conjugated labels in the membrane pores, we also optimized the nitrocellulose membrane capillary flow time (Figure 3b) [48]. To do so, nitrocellulose membranes from different suppliers (Millipore^®^, Sartorius, and Cytiva^TM^) with diverse flow times were tested. A selection of the best-performing membranes in half-strip assays, with flow times between 75 and 200 s/4 cm, were evaluated in sample buffer and RT-qPCR negative nasopharyngeal swabs. Once again, significant differences were only observed when nasopharyngeal samples were tested. The Cytiva^TM^ Whatman Immunopore RP membrane showed significantly more invalid results than the rest of the tested alternatives. Since the incidence of false positive results was minimized when using the HFC135 Millipore^®^ Hi-Flow Plus membrane, we chose this material to work with in further experiments.

**Figure 4:**
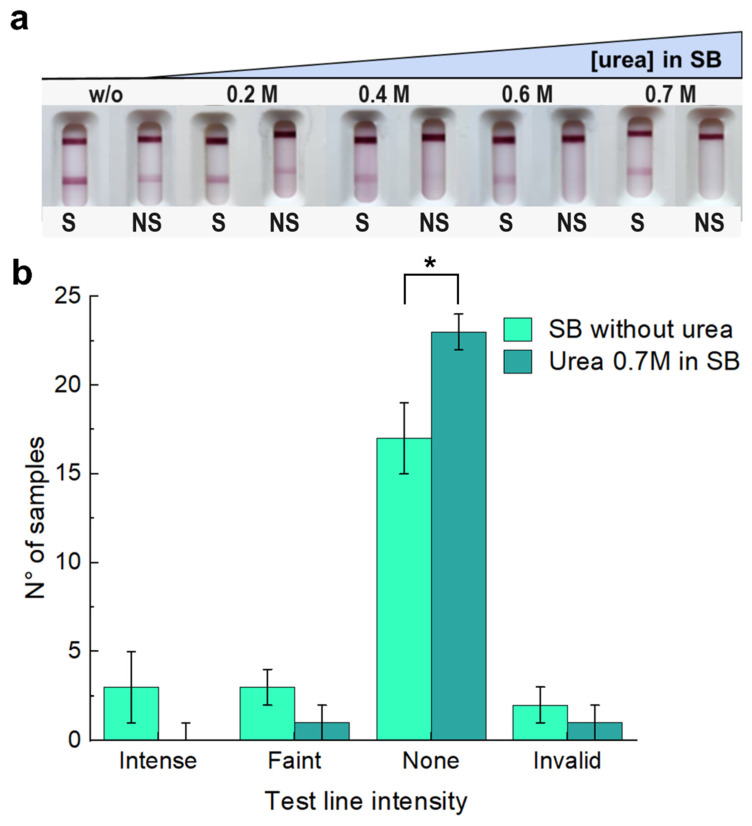
(**a**) Full strip results using a spiked (inactivated virus, Ct = 26) and non-spiked (sample buffer) RT-qPCR nasopharyngeal swab sample, which previously showed a high non-specific signal, with increasing concentration of urea in sample buffer. (**b**) Sample buffer with and without 0.7 M urea addition is compared using full strips in spiked and non-spiked RT-qPCR nasopharyngeal swab samples. The results show a decrease in the non-specific signal for most of the NS RT-qPCR nasopharyngeal swab samples evaluated. * *p* value of 0.019. In all cases, the strips are dispensed with 2 µg per strip of horse anti-N in the test line on a Millipore Hi-Flow Plus HFC13502 membrane with 2% BSA in the blocking buffer. The signal intensity on the TL was qualitatively measured by the naked eye by three independent users. The error bars were then estimated by SD of these three independents measurements.

Next, we evaluated the sample buffer as another parameter to optimize for NSB reduction. After evaluating different buffering alternatives (varying both salt identity and pH values) and BSA concentrations in the sample buffer (Discussion S1, SI) with no significant effects over the degree of observed NSB (Figure S10a,b), we turned to studying the influence of adding urea to the sample buffer (Figure 4). Urea has been widely used to differentiate true-positive from false-positive antibody results in ELISA and LFT [59,60,61]; therefore, we rationalized that urea can impact nonspecific hydrophobic forces that may be responsible for NSB [62,63,64,65,66]. To test this idea, we first evaluated a particular nasopharyngeal swab sample that displayed a strong non-specific signal in the absence of viral particles with the optimized test strips in sample buffers containing increasing urea concentrations (Figure 4a). A gradual decrease in the non-specific signal seen in the non-spiked sample could be observed as the urea concentration was increased, and it was no longer seen at 0.7 M urea. This promising result prompted us to compare the same lot of 25 nasopharyngeal swabs using sample buffers without urea and with 0.7 M urea (Figure 4b). We observed that the addition of 0.7 M urea eliminated all the false positive results except for one sample, which still gave a faint non-specific signal. We continued the optimization with 0.7 M urea in the sample buffer.

Finally, the last parameter that we studied to reduce NSB was the conjugate. To study if the conjugate was the cause of possible artifact generation producing NSB, we carried out a conjugate pad transplantation experiment [35]. The observed results indicated that NSB was not detectable in our test strips when a commercial conjugate pad was used, even in the absence of urea (Figure S11). Thus, we turned to optimizing the blocking protocol following the Ab physisorption, consisting initially of a first blocking step with 0.05% *w*/*v* PEG 20000 and a second blocking with 1% *w*/*v* BSA. Insufficient blocking may lead to non-specific interactions between the bare AuNPs surface and proteins from nasal samples, while excessive blocking can lead to protein aggregation on the surface of the AuNPs, which may in turn induce NSB [56]. Additionally, we also optimized the conjugate concentration, as it simultaneously affects both sensitivity (insufficient label concentration can lead to low signal intensity) and NSB (high concentration can result in NSB and background noise) [67]. Table S2 details the distinct characteristics of the seven tested prototypes. In short, the conjugate was dispensed either at OD 15 or OD 10 and blocked following different BSA and PEG concentrations. This optimization allowed us to decrease the rate of false positives to less than 2% and invalid tests below 6% determined with a prototype in which the conjugate was blocked only with 0.5% BSA and dispensed at OD 10 (Figure S12).

### 3.4. Limit of Detection and Evaluation in Real Setting

**Figure 5:**
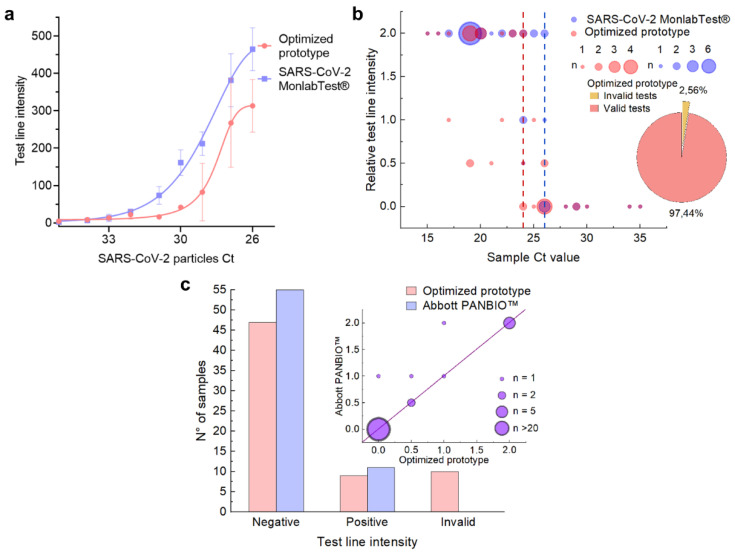
(**a**) Limit of detection (LOD) comparison between the optimized prototype (red) and the MonlabTest^®^ (blue) using negative nasal swab pools spiked with dilutions of SARS-CoV-2 inactivated particles. The test line intensities are plotted against each sample’s Ct value. (**b**) Optimized prototype performance on RT-qPCR positive nasopharyngeal swabs (red) compared with the commercial MonlabTest^®^ (blue). The relative test line intensities are plotted against each sample’s Ct value. Dotted lines mark the lowest Ct value at which each test failed to detect a RT-qPCR positive sample. The pie chart on the right shows the proportion of valid (red) and invalid (yellow) tests displayed by the optimized prototype. (**c**) Optimized prototype performance on nasal swabs (red) compared with the Abbott PANBIO™ commercial test (blue). The graphic shows the number of positive, negative, and invalid samples for both tests. The inset on the right shows the comparison of the relative test line intensities observed with both prototypes.

To compare the performance of the optimized prototype with commercial tests, we first determined the LOD with spiked nasal swabs for the chosen prototype and a commercial test. For this experiment, fresh nasal swabs of known negative individuals were collected in the selected sample buffer and pooled in three distinct samples, which served as biological replicates. Dilutions of inactivated SARS-CoV-2 particles with a Ct = 23 were prepared in each of these pools and ran in the LFA. Next, the LOD was calculated using a low-concentration dilution [34]. While the intensity of the test line was higher in the commercial tests, the LOD was Ct = 32 for the prototype reported here and Ct = 33 for the commercial test, in agreement with the manufacturer’s value (Figure 5a). Thus, qualitatively, we can conclude that in terms of LOD and NSB, our locally produced and developed tests’ performance is comparable to commercial tests.

To further characterize the performance of our tests as compared to commercial alternatives, we performed a validation in two different settings. First, we evaluated the performance compared with commercial tests (SARS-CoV-2 MonlabTest^®^) in nasopharyngeal swabs positive for SARS-CoV-2 as determined by RT-qPCR. In this case, the assays were performed in a BSL3 facility; thus, the TL intensity was determined qualitatively. The lowest Ct value at which each test starts to fail to detect an RT-qPCR positive sample was a Ct = 24 for COVIDAR-Ag and Ct = 26 for the commercial antigen test (Figure 5b, indicated by a dotted line), which is consistent with the small difference in LOD determined in the previous experiment.

Next, we evaluated our prototype on fresh nasal swabs samples, and their performance was also compared to another commercially available rapid antigen test (Abbott PANBIO™ COVID-19-Ag rapid test) (Figure 5c). Our results on samples from volunteers with symptoms compatible with COVID-19 in the UNQ COVID-19 Emergency Diagnostics Unit showed that while most of the samples were negative, the degree of accuracy when compared to RT-qPCR was comparable between our prototype and the commercial one. With these assays, we estimated the performance of our prototype in terms of sensitivity, specificity [68], positive predictive value (PPV), negative predictive value (NPV) [69], and *κ* value [70] using fresh nasal samples (Table 2). Our prototype reached a sensitivity of 90.9%. The main difference in performance was due to the invalid tests, which suggests that further optimization of the control line to bind more tightly to horse conjugates may be beneficial [71]. To further evaluate if our prototype followed WHO recommendations on minimum performance requirements for Ag-RDTs (≥80% sensitivity and ≥97% specificity compared to a nucleic acid amplification test in suspected COVID-19 cases [72,73]), we estimated the performance with the data of positive and negative tests presented in Figure 5b and Figure S12 (prototype 5). While a more definitive validation is required to commercialize this prototype as is, the parameters obtained were in good agreement with the WHO recommendations (Table 2).

## 4. Conclusions

A functional rapid LFT prototype for SARS-CoV-2 antigen detection based on its nucleocapsid protein was developed and tested in real samples using locally produced horse pAbs as the main critical resource. These antibodies outperformed commercially available mAbs, and the test’s overall performance followed WHO recommendations and was comparable to off-the-shelf LFTs in terms of sensitivity and specificity. As expected, the development process faced several challenges, and multiple trial-and-error re-optimization steps were required. The result of this optimization is included as part of this report to facilitate the local development of these tests based on other polyclonal antibodies or recognition elements that may be locally available in other settings.

Our results clearly illustrate the importance of developing and optimizing a purification protocol for the antibodies used as recognition elements that can be directly evaluated in the context of the LFA. In this regard, to reach an acceptable performance for both horse and llama pAbs, a two-step procedure involving caprylic acid precipitation and affinity purification using N protein-coated columns was required. Of course, this modification introduces additional costs; however, these viral proteins can be readily obtained recombinantly in *E. coli* without the requirement of expensive media. For cost estimation of the tests performed with affinity purified pAbs, both the affinity purification and the hyperimmune plasma need to be considered. On the one hand, the N protein-coated column used here required 20 mg of protein, which can be reused to obtain at least 200 mg of pAbs. This column had a production cost of USD 200, USD 1/mg. On the other hand, given a yield of 1 mg per 2 mL of hyperimmune plasma after caprylic acid precipitation, the cost of 1 mg pAbs before purification is USD 1. Thus, the total cost of 1 mg of affinity-purified pAbs, which yields 500 tests, is around USD 2, with a lower cost than most commercial antibodies. Thus, we thought that our assay may be cost effective, particularly in an outbreak setting when no other resources are available.

Furthermore, our results suggest that the performance of the test and the optimization of the running conditions is only effective when performed on real samples, as most of the sensitivity and specificity depend on the interference present in nasal and nasopharyngeal swab samples. While this may be obvious for most experts in the field, it remains true that the bottleneck of new POC test development arises long after publication, when real samples are tested. We think that our results should encourage the community to enhance the strategies to facilitate testing early developments with safe and anonymous real samples that properly represent the analytical challenge antigen test development.

We expect that this report contributes to take advantage of the efforts carried out to develop and optimize pAb and mAb production processes at a local level for the development of antigen tests [4,67,74,75,76,77]. This will not only allow the local healthcare community to be better prepared for possible urgent outbreaks in terms of treatment but also in terms of POC diagnostics. We hope that this serves as a resource to encourage local and open-access development, especially in LMICs, as a tool for fighting the inherent inequalities posed by the higher production and importation abilities of richer countries. After all, reducing the spread of contagious diseases in emergencies would have a global benefit.

Addressing the supply chain issues in LMICs and building capacity for the domestic manufacture of essential diagnostic tools like LFTs is crucial. This would not only improve preparedness and response during health emergencies but also allow these regions greater autonomy in managing their public health needs.

## Figures and Tables

**Table 1:** Set of tested and selected parameters.

LFT Element	Parameter	Tested Variables	Selected
Sample	Antigenic protein	Spike and nucleocapsid	Nucleocapsid
	Sample buffer composition	PBS w/Tween 20 or Triton x-100 between 0.5 and 4% w/ and w/o urea between 0.2 and 0.7 M	PBS w/1% Triton x-100 and urea 0.7 M
	Sample pad pre-treatment	PBS w/1% Triton x-100 and w/ or w/o urea between 0.7 and 7.2 M	PBS w/1% Triton x-100 and urea 2 M
Membrane	Test line (TL) Ab	Horse and llama pAbs, mouse mAb	Horse pAb
	TL dispensing buffer	PBS pH 7.4, Tris-Glycine pH 7, MES 25 mM pH 6.5	MES 25 mM, pH 6.5
	Control line	Proteins A and G	Protein A 0.36 µg
	Nitrocellulose membrane	Capillary flow times between 75 and 200 s/4 cm	HFC13502 (Millipore)
	Blocking	PBS w/3% sucrose and BSA between 0.15 and 6%	PBS w/3% sucrose and 2% BSA
Conjugate	Antibody	Horse pAb, mouse mAb, commercial rabbit mAb	Horse pAb
	Conjugation pH	5, 6, 7, 8	7
	Ab concentration	40, 80, 160, 240, 320 ng/µL	80
	Blocking	BSA and PEG 20,000, varying amounts	0.5% BSA, no PEG
	OD	5, 10, 15	10

**Table 2:** Performance of the optimized prototype.

	Optimized Prototype vs. Abbott PANBIO™	Optimized Prototype vs. RT-qPCR
Sensitivity% (CI 95%)	90.9 (62.3–99.5)	83.3 (68.1–92.3)
Specificity% (CI 95%)	100.0 (93.5–100.0)	100.0 (93.0–100.0)
PPV% (CI 95%)	100.0 (72.3–100.0)	100.0 (88.7–100.0)
NPV% (CI 95%)	98.2 (90.6–99.9)	89.5 (78.9–95.1)
*κ* value (CI 95%)	0.94 (83.3–100.0) Almost perfect agreement [70]	0.85 (74.3–96.6) Almost perfect agreement [70]

## Data Availability

Data are contained within the article or Supplementary Materials.

## Supplementary Materials

The following supporting information can be downloaded at: https://www.mdpi.com/article/10.3390/bios14090416/s1, Figure S1: (a) Image taken by SEM of a 1:10 dilution of the AuNP seeds, where the spherical shape of the seeds is observed; (b) Image taken by SEM of an undiluted solution of grown AuNPs, where the shape is observed to be a little less spherical compared to the seeds.; (c) Spectra obtained by UV-vis measurement. The red curve corresponds to the 20 nm AuNPs, whose maximum absorbance peak is 519 nm and corresponds to 1.16 absorbance units. The red curve corresponds to the 30 nm AuNPs for which a change in the spectrum is observed towards longer wavelengths, as corresponds to larger sizes of the AuNPs. In this case the maximum absorbance peak is 525 nm and corresponds to 1.3 absorbance units; (d) Histograms with the frequency of AuNP seed sizes (red), and grown AuNP sizes (blue), showing a normal distribution. The average size is 21 nm, with a standard deviation of 2 nm for the seeds and 31 nm, with a 3 nm standard deviation for the grown AuNPs. Comparing both histograms, it can be seen the effective growth of the AuNPs; (e) Spectra obtained by UV-vis measurement. The blue curve corresponds to 31 nm AuNPs, whose maximum absorbance peak is 525 nm; Figure S2: Full strip final conformation with 60 mm × 3.7 mm dimensions placed inside the commercial LFT cassette (Zhuhai Ideal Plastic Product Co., Ltd., Zhuhai City, China. A 25 mm × 300 mm Millipore HFC13502 nitrocellulose membrane was assembled on a 60 mm × 300 mm backing card from DCN Diagnostics, on the corresponding section marked for this purpose; Figure S3: Dose-response relationship curve obtained from ELISA sandwich assays using plates sensitized with horse polyclonal anti-RBD pAb (at 50 ng per well, at 4 °C ON). In the second step of the assay, the plates were incubated with (a) the ectodomain of the SARS-CoV-2 spike protein at 37 °C for 1 h or (b) VSV pseudotyped with SARS-CoV-2 spike. The assays were carried out in PBS solution (blue) and PBS-1% Tween 20 (red). Detection was first performed with mouse monoclonal anti-spike as the primary antibody (at 65 µg/mL, at 37 °C for 1 h) and then with commercial HRP-conjugated Abs (goat anti-mouse-HRP, R&D Systems, Inc., United States), according to the manufacturer’s instructions, measuring the optical density of the samples at 450 nm. The detection limit (indicated with a dotted line) was obtained from the upper limit of the IC95 of the fit to a rectangular hyperbola (solid line) evaluated at (a) 0.06 ng/100 µL and (b) 800 copies of VSV/100 µL; (c) Full strip assay results comparing S and N protein detection using sample buffer spiked with these proteins. The S protein detection strips (top panel) were dispensed with 1 µg of horse pAbs anti-RBD and a conjugate using the same Ab; Figure S4: (a) SDS-PAGE of horse pAbs purification process. (1) Molecular weight marker Precision plus protein standard (BioRad); (2) Supernatant after caprylic acid precipitation; (3) N protein affinity column flow-through; (4) PBS buffer wash; (5) Purified fraction (Tris-Gly pH 7); (6) Purified fractions pool; (7) Glycine buffer wash; (b) SDS-PAGE of llama pAbs purification process. (1) BSA pattern; (2) Unpurified llama sera; (3) Supernatant after caprylic acid precipitation; (4) N protein affinity column flow-through; (5–8) Purified fractions (Tris-Gly pH 7); (c) SDS-PAGE of mouse mAbs protein G purification process. 1) Mouse ascitic fluid (1:10 dilution); (2–6) Purified fractions (Tris-Gly pH 7); (7–8) Protein G column flow-through; 9) Molecular weight marker Precision plus protein standard (BioRad); Figure S5: (a) Half-strip assay results using sample buffer with (S) and without (NS) inactivated virus (Ct = 26) with three different anti-N conjugates: horse pAb, mouse mAb, and commercial rabbit mAb anti-SARS-CoV-2 NP, clon NJ1, Creative Diagnostics. In all three cases, an intense signal can be seen on the TL, stronger in the case of horse conjugate. A faint non-specific signal can be seen on the TL on the rabbit conjugate’s NS assay, indicating a possible NSB problem with this particular conjugate. In all cases, the strips were dispensed with 1 µg horse anti-N per strip on the test line on a Sartorius UniSart CN95 membrane; (b) Histograms showing the relative intensity (qualitatively) results for full-strip assays using spiked (S, inactivated virus particles) or non-spiked (NS, deionized water) RT-qPCR negative nasopharyngeal swab samples with three different anti-N conjugates: horse pAb, mouse mAb, and commercial rabbit mAb anti-SARS-CoV-2 NP, clon NJ1, Creative Diagnostics; Figure S6: Histograms showing the performance of full-strip assays using horse conjugates under various conditions, as indicated by the relative intensity of the test line; Figure S7: (a) Histograms showing half-strip assay results using two different detergents, Tween 20 and Triton x-100 in sample buffer comparison. Sample buffer was tested by adding various concentrations of N protein and inactivated SARS-CoV-2 dilutions from a Ct = 23 stock, or without any addition. N protein spiked buffer showed a higher signal for the assays with Tween 20. This could be attributed to the fact that Triton x-100 may affect the stability of the recombinant protein. Both signals are comparable when an excess of N protein is added. On the other hand, in virus-spiked spiked buffer, a considerably higher signal is observed when Triton x-100 is used, which may indicate that this detergent functions better for exposing the antigen; (b) Histograms showing half-strip assay results obtained using increasing concentrations of Triton x-100 in the sample buffer comparison; Figure S8: (a) Half-strip assay results for horse pAbs dispensed in different buffers, Tris-Glycine buffer pH 7, obtained from post affinity purification column (elution in glycine buffer 0.1 M pH 2.8 and collected in 10% Tris buffer 1 M pH 8), PBS pH 7.4, and MES 25 mM pH 6.5. Sample buffer (PBS + Triton x-100 1%) was tested with (Spiked (S)) or without (Non-spiked (NS) 10 ng of N protein. N protein spiked buffer showed a strong signal in the three cases. However, in non-spiked cases, a faint signal can be seen for both Tris-Glycine and PBS buffers, but no signal can be seen with MES buffer. For all cases, strips were dispensed by hand with 1 µg horse anti-N per strip on the test line on a Sartorius UniSart CN95 membrane and using AuNPs-horse pAb conjugate; (b) Histograms showing the relative intensity (qualitatively) results for full strip assays with the test line dispensed in PBS or MES buffer using non-spiked RT-qPCR negative nasopharyngeal samples; Figure S9:. Full strip assays using spiked (S, inactivated virus, Ct = 26) and non-spiked (NS, deionized water) RT-qPCR nasopharyngeal swab samples ran in UniSart CN95 membranes previously blocked with increasing amounts of BSA. In all cases, the strips’ test lines were dispensed with 2 µg horse anti-N per strip; Figure S10: (a) Full-strip assay results using sample buffers with different molarities, pH ranges, and BSA and detergent concentrations tested with non-spiked RT-qPCR negative nasopharyngeal swab sample that displayed a strong non-specific signal in the absence of viral particles with the optimized test strips: (1) Sodium borate 10 mM, pH 10 + NaCl 1.37 M + BSA 0.02% + Tween 20 0.5%; (2) Sodium borate 10 mM, pH 10 + BSA 0.02% + Tween 20 0.5%; (3) Sodium carbonate 10 mM, pH 11 + NaCl 1.37 M + BSA 0.02% + Tween 20 0.5%; (4) Sodium carbonate 10 mM, pH 10 + BSA 0.02% + Tween 20 0.5%; (5) TBS pH 7.5 + BSA 0.02% + Tween 20 0.5%; (6) Tricine 100 mM, pH 7.5 + NaCl 100 mM + Tween 20 1%; (7) PBS pH 7.2 + BSA 0.2% + Tween 20 0.5%; (8) Sodium borate 100 mM, pH 8 + BSA 0.02% + Tween 20 0.5%; (9) Sodium borate 100 mM, pH 8 + BSA 0.2% + Tween 20 1%; (10) TBS pH 8.2 + BSA 0.2% + Tween 20 1%; (11) Tricine 100 mM, pH 8.4 + Tween 20 1%; (12) PBS pH 7.2 + Tritón 1%. Strips were dispensed with 2 µg horse anti-N in MES 25 mM pH 6.5 per strip on the test line on a Millipore HFC13502 membrane and using AuNPs-horse pAb conjugate. A range of test line intensities can be observed in all cases, with the lowest intensity observed for buffers 2, 5, and 12; (b) Histograms showing the relative intensity (qualitatively) results for full-strip assays using spiked (S, inactivated virus particles) or non-spiked (NS, deionized water) RT-qPCR negative nasopharyngeal swab samples with the three previous selected sample buffers: Sodium borate 10 mM, pH 10 + BSA 0.02% + Tween 20 0.5%, TBS pH 7.5 + BSA 0.02% + Tween 20 0.5% and, PBS pH 7.2 + Tritón 1%; Figure S11: Full-strip assay results from 6 representative nasopharyngeal swabs out of 15 tested with commercial tests (Artron COVID-19 Rapid Antigen Test Kit), COVIDAR-Ag conjugate pad transplantation to a commercial test, and commercial conjugate pad transplantation to the COVIDAR-Ag test presented here (from top to bottom); Figure S12: Histograms showing the relative amounts of negative, invalid, and false positive results obtained using the different conjugate pad prototypes are summarized in Table S2; Table S1: Conjugation parameters tested and selected for each antibody; Table S2: Conjugate pad prototypes.

## References

[1] Budd J., Miller B.S., Weckman N.E., Cherkaoui D., Huang D., Decruz A.T., Fongwen N., Han G.-R., Broto M., Estcourt C.S. (2023). Lateral Flow Test Engineering and Lessons Learned from COVID-19. Nat. Rev. Bioeng..

[2] BinaxNOWTM COVID-19 Ag Card (2020). FDA. https://www.fda.gov/media/141570/download.

[3] Parolo C., Sena-Torralba A., Bergua J.F., Calucho E., Fuentes-Chust C., Hu L., Rivas L., Álvarez-Diduk R., Nguyen E.P., Cinti S. (2020). Tutorial: Design and Fabrication of Nanoparticle-Based Lateral-Flow Immunoassays. Nat. Protoc..

[4] Grant B.D., Anderson C.E., Alonzo L.F., Garing S.H., Williford J.R., Baughman T.A., Rivera R., Glukhova V.A., Boyle D.S., Dewan P.K. (2021). A SARS-CoV-2 Coronavirus Nucleocapsid Protein Antigen-Detecting Lateral Flow Assay. PLoS ONE.

[5] Pucca M.B., Cerni F.A., Janke R., Bermúdez-Méndez E., Ledsgaard L., Laustsen A.H. (2019). History of Envenoming Therapy and Current Perspectives. Front. Immunol..

[6] Potet J., Beran D., Ray N., Alcoba G., Habib A.G., Iliyasu G., Waldmann B., Ralph R., Faiz M.A., Monteiro W.M. (2021). Access to Antivenoms in the Developing World: A Multidisciplinary Analysis. Toxicon: X.

[7] Chippaux J.-P., Massougbodji A., Habib A.G. (2019). The WHO Strategy for Prevention and Control of Snakebite Envenoming: A Sub-Saharan Africa Plan. J. Venom. Anim. Toxins Incl. Trop. Dis..

[8] Gutiérrez J.M. (2019). Global Availability of Antivenoms: The Relevance of Public Manufacturing Laboratories. Toxins.

[9] Fan H.W., Vigilato M.A.N., Pompei J.C.A., Gutiérrez J.M., Red de Laboratorios Públicos Productores de Antivenenos de América Latina (RELAPA) (2019). Situación de Los Laboratorios Públicos Productores de Antivenenos En América Latina. Rev. Panam. Salud Publica.

[10] Temprano G., Angeleri P., Dokmetjian J.C. (2017). La Producción Pública de Antivenenos En La Región de Las Américas Como Factor Clave En Su Accesibilidad. Rev. Panam. Salud Publica.

[11] Byzova N.A., Urusov A.E., Zherdev A.V., Dzantiev B.B. (2018). Multiplex Highly Sensitive Immunochromatographic Assay Based on the Use of Nonprocessed Antisera. Anal. Bioanal. Chem..

[12] Cantera J.L., Cate D.M., Golden A., Peck R.B., Lillis L.L., Domingo G.J., Murphy E., Barnhart B.C., Anderson C.A., Alonzo L.F. (2021). Screening Antibodies Raised against the Spike Glycoprotein of SARS-CoV-2 to Support the Development of Rapid Antigen Assays. ACS Omega.

[13] Gallardo-Alfaro L., Lorente-Montalvo P., Cañellas M., Carandell E., Oliver A., Rojo E., Riera B., Llobera J., Bulilete O., Leiva A. (2023). Diagnostic Accuracy of Panbio^TM^ Rapid Antigen Test for SARS-CoV-2 in Paediatric Population. BMC Pediatr..

[14] Ojeda D.S., Ledesma M.M.G.L., Pallarés H.M., Navarro G.S.C., Sanchez L., Perazzi B., Villordo S.M., Alvarez D.E., Group B.W., Echavarria M. (2021). Emergency Response for Evaluating SARS-CoV-2 Immune Status, Seroprevalence and Convalescent Plasma in Argentina. PLoS Pathog..

[15] Torres C., Mojsiejczuk L., Acuña D., Alexay S., Amadio A., Aulicino P., Debat H., Fay F., Fernández F., Giri A.A. (2021). Cost-Effective Method to Perform SARS-CoV-2 Variant Surveillance: Detection of Alpha, Gamma, Lambda, Delta, Epsilon, and Zeta in Argentina. Front. Med..

[16] Grant B.D., Anderson C.E., Williford J.R., Alonzo L.F., Glukhova V.A., Boyle D.S., Weigl B.H., Nichols K.P. (2020). SARS-CoV-2 Coronavirus Nucleocapsid Antigen-Detecting Half-Strip Lateral Flow Assay Toward the Development of Point of Care Tests Using Commercially Available Reagents. Anal. Chem..

[17] Baker A.N., Richards S.-J., Guy C.S., Congdon T.R., Hasan M., Zwetsloot A.J., Gallo A., Lewandowski J.R., Stansfeld P.J., Straube A. (2020). The SARS-COV-2 Spike Protein Binds Sialic Acids and Enables Rapid Detection in a Lateral Flow Point of Care Diagnostic Device. ACS Cent. Sci..

[18] Salcedo N., Reddy A., Gomez A.R., Bosch I., Herrera B.B. (2022). Monoclonal Antibody Pairs against SARS-CoV-2 for Rapid Antigen Test Development. PLoS Neglected Trop. Dis..

[19] Harvey W.T., Carabelli A.M., Jackson B., Gupta R.K., Thomson E.C., Harrison E.M., Ludden C., Reeve R., Rambaut A., Peacock S.J. (2021). SARS-CoV-2 Variants, Spike Mutations and Immune Escape. Nat. Rev. Microbiol..

[20] Guruprasad L. (2021). Human SARS CoV-2 Spike Protein Mutations. Proteins: Struct. Funct. Bioinform..

[21] Das D., Kammila S., Suresh M.R. (2010). Development, Characterization, and Application of Monoclonal Antibodies against Severe Acute Respiratory Syndrome Coronavirus Nucleocapsid Protein. Clin. Vaccine Immunol..

[22] Rak A., Gorbunov N., Kostevich V., Sokolov A., Prokopenko P., Rudenko L., Isakova-Sivak I. (2023). Assessment of Immunogenic and Antigenic Properties of Recombinant Nucleocapsid Proteins of Five SARS-CoV-2 Variants in a Mouse Model. Viruses.

[23] Bai Z., Cao Y., Liu W., Li J. (2021). The SARS-CoV-2 Nucleocapsid Protein and Its Role in Viral Structure, Biological Functions, and a Potential Target for Drug or Vaccine Mitigation. Viruses.

[24] Zhang L., Fang X., Liu X., Ou H., Zhang H., Wang J., Li Q., Cheng H., Zhang W., Luo Z. (2020). Discovery of Sandwich Type COVID-19 Nucleocapsid Protein DNA Aptamers. Chem. Commun..

[25] Gonzalez Lopez Ledesma M.M., Sanchez L., Ojeda D.S., Oviedo Rouco S., Rossi A.H., Varese A., Mazzitelli I., Pascuale C.A., Miglietta E.A., Rodríguez P.E. (2022). Longitudinal Study after Sputnik V Vaccination Shows Durable SARS-CoV-2 Neutralizing Antibodies and Reduced Viral Variant Escape to Neutralization over Time. mBio.

[26] Al Kaabi N., Zhang Y., Xia S., Yang Y., Al Qahtani M.M., Abdulrazzaq N., Al Nusair M., Hassany M., Jawad J.S., Abdalla J. (2021). Effect of 2 Inactivated SARS-CoV-2 Vaccines on Symptomatic COVID-19 Infection in Adults. JAMA.

[27] Hadj Hassine I. (2022). Covid-19 Vaccines and Variants of Concern: A Review. Rev. Med. Virol..

[28] Gomes M.P.d.B., Linhares J.H.R., dos Santos T.P., Pereira R.C., Santos R.T., da Silva S.A., Souza M.C.d.O., da Silva J.F.A., Trindade G.F., Gomes V.S. (2023). Inactivated and Immunogenic SARS-CoV-2 for Safe Use in Immunoassays and as an Immunization Control for Non-Clinical Trials. Viruses.

[29] Brizuela M.E., Goñi S.E., Cardama G.A., Zinni M.A., Castello A.A., Sommese L.M., Farina H.G. (2022). Correlation of SARS-CoV-2 Viral Load and Clinical Evolution of Pediatric Patients in a General Hospital From Buenos Aires, Argentina. Front. Pediatr..

[30] González Viacava M.B., Varese A., Mazzitelli I., Lanari L., Ávila L., García Vampa M.J., Geffner J., Cascone O., Dokmetjian J.C., de Roodt A.R. (2022). Immune Maturation Effects on Viral Neutralization and Avidity of Hyperimmunized Equine Anti-SARS-CoV-2 Sera. Antibodies.

[31] De Simone E.A., Saccodossi N., Ferrari A., Leoni J. (2008). Development of ELISAs for the Measurement of IgM and IgG Subclasses in Sera from Llamas (*Lama glama*) and Assessment of the Humoral Immune Response against Different Antigens. Vet. Immunol. Immunopathol..

[32] Nudel B.C., Perdoménico C., Iácono R., Cascone O. (2012). Optimization by Factorial Analysis of Caprylic Acid Precipitation of Non-Immunoglobulins from Hyperimmune Equine Plasma for Antivenom Preparation. Toxicon.

[33] Kimling J., Maier M., Okenve B., Kotaidis V., Ballot H., Plech A. (2006). Turkevich Method for Gold Nanoparticle Synthesis Revisited. J. Phys. Chem. B.

[34] Armbruster D.A., Pry T. (2008). Limit of Blank, Limit of Detection and Limit of Quantitation. Clin. Biochem. Rev..

[35] Patriquin G., Davidson R.J., Hatchette T.F., Head B.M., Mejia E., Becker M.G., Meyers A., Sandstrom P., Hatchette J., Block A. (2021). Generation of False-Positive SARS-CoV-2 Antigen Results with Testing Conditions Outside Manufacturer Recommendations: A Scientific Approach to Pandemic Misinformation. Microbiol. Spectr..

[36] Jacot D., Greub G., Jaton K., Opota O. (2020). Viral Load of SARS-CoV-2 across Patients and Compared to Other Respiratory Viruses. Microbes Infect..

[37] Huang C.-G., Lee K.-M., Hsiao M.-J., Yang S.-L., Huang P.-N., Gong Y.-N., Hsieh T.-H., Huang P.-W., Lin Y.-J., Liu Y.-C. (2020). Culture-Based Virus Isolation To Evaluate Potential Infectivity of Clinical Specimens Tested for COVID-19. J. Clin. Microbiol..

[38] Peng T., Dong L., Feng X., Yang Y., Wang X., Niu C., Liang Z., Qu W., Zou Q., Dai X. (2023). Relationship between SARS-CoV-2 Nucleocapsid Protein and *N* Gene and Its Application in Antigen Testing Kits Evaluation. Talanta.

[39] GeneTex SARS-CoV-2 (COVID-19) Nucleocapsid Antibody. https://www.genetex.com/Product/Detail/SARS-CoV-2-COVID-19-Nucleocapsid-antibody/GTX135357?srsltid=AfmBOoqhuSrW0yt-KPo8-vy81qvpQH2WDl1sKzvoxnYZsUWTcAT9oif8#references.

[40] CD—Creative Diagnostics—Human Anti-SARS-CoV-2 Nucleoprotein Monoclonal Antibody for ELISA. https://www.creative-diagnostics.com/sars-cov-2-nucleoprotein-antibody-277914-144.htm.

[41] GeneScript—MonoRabTM SARS-CoV-2 Nucleocapsid Antibody (N34), mAb, Rabbit. https://www.genscript.com/antibody/A02136-MonoRab_SARS_CoV_2_Nucleocapsid_Antibody_N34_mAb_Rabbit.html?_gl=1*16y79ep*_up*MQ..&gclid=Cj0KCQjw2ou2BhCCARIsANAwM2FDWuTFnTzJ7T1Au-ZS7HGCIVjoF__HgD8wll-0NJkmxdWBlceb8ZQaAkcVEALw_wcB.

[42] Zhang Y., Juhas M., Kwok C.K. (2023). Aptamers Targeting SARS-COV-2: A Promising Tool to Fight against COVID-19. Trends Biotechnol..

[43] Huang Y., Chen X., Zhang J., Tian W., Liu S., Chun-Yee Tam R., Yang C., Song Y. (2023). Aptamer-Based Strategies against SARS-CoV-2 Viruses. BMEMat.

[44] Chakraborty B., Das S., Gupta A., Xiong Y., Vyshnavi T.-V., Kizer M.E., Duan J., Chandrasekaran A.R., Wang X. (2022). Aptamers for Viral Detection and Inhibition. ACS Infect. Dis..

[45] Dong Y., Wang J., Chen L., Chen H., Dang S., Li F. (2024). Aptamer-Based Assembly Systems for SARS-CoV-2 Detection and Therapeutics. Chem. Soc. Rev..

[46] Yang M., Li C., Ye G., Shen C., Shi H., Zhong L., Tian Y., Zhao M., Wu P., Hussain A. (2024). Aptamers Targeting SARS-CoV-2 Nucleocapsid Protein Exhibit Potential Anti Pan-Coronavirus Activity. Signal Transduct. Target. Ther..

[47] Poolsup S., Zaripov E., Hüttmann N., Minic Z., Artyushenko P.V., Shchugoreva I.A., Tomilin F.N., Kichkailo A.S., Berezovski M.V. (2023). Discovery of DNA Aptamers Targeting SARS-CoV-2 Nucleocapsid Protein and Protein-Binding Epitopes for Label-Free COVID-19 Diagnostics. Mol. Ther.—Nucleic Acids.

[48] Zhan L., Granade T., Liu Y., Wei X., Youngpairoj A., Sullivan V., Johnson J., Bischof J. (2020). Development and Optimization of Thermal Contrast Amplification Lateral Flow Immunoassays for Ultrasensitive HIV P24 Protein Detection. Microsyst. Nanoeng..

[49] Liu Y., Zhan L., Qin Z., Sackrison J., Bischof J.C. (2021). Ultrasensitive and Highly Specific Lateral Flow Assays for Point-of-Care Diagnosis. ACS Nano.

[50] Hagström A.E.V., Garvey G., Paterson A.S., Dhamane S., Adhikari M., Estes M.K., Strych U., Kourentzi K., Atmar R.L., Willson R.C. (2015). Sensitive Detection of Norovirus Using Phage Nanoparticle Reporters in Lateral-Flow Assay. PLoS ONE.

[51] Kim D.S., Kim Y.T., Hong S.B., Kim J., Heo N.S., Lee M.-K., Lee S.J., Kim B.I., Kim I.S., Huh Y.S. (2016). Development of Lateral Flow Assay Based on Size-Controlled Gold Nanoparticles for Detection of Hepatitis B Surface Antigen. Sensors.

[52] Martinez-Liu C., Machain-Williams C., Martinez-Acuña N., Lozano-Sepulveda S., Galan-Huerta K., Arellanos-Soto D., Meléndez-Villanueva M., Ávalos-Nolazco D., Pérez-Ibarra K., Galindo-Rodríguez S. (2022). Development of a Rapid Gold Nanoparticle-Based Lateral Flow Immunoassay for the Detection of Dengue Virus. Biosensors.

[53] Duong N.-D., Nguyen-Phuoc K.-H., Mai-Hoang T.-D., Do K.-Y.T., Huynh T.-B., Nguyen N.-T.T., Tran T.L., Tran-Van H. (2022). Fabrication of Lateral Flow Immunoassay Strip for Rapid Detection of Acute Hepatopancreatic Necrosis Disease. 3 Biotech.

[54] Trakoolwilaiwan T., Takeuchi Y., Leung T.S., Sebek M., Storozhuk L., Nguyen L., Tung L.D., Thanh N.T.K. (2023). Development of a Thermochromic Lateral Flow Assay to Improve Sensitivity for Dengue Virus Serotype 2 NS1 Detection. Nanoscale.

[55] Abousenna M.S., Sayed R.H., Shaimaa A.E., Shasha F.A., El Sawy S.E.A., Darwish D.M. (2024). Sensitivity of Lateral Flow Technique for Diagnosis of Canine Parvovirus. Sci. Rep..

[56] de Puig H., Bosch I., Carré-Camps M., Hamad-Schifferli K. (2017). Effect of the Protein Corona on Antibody–Antigen Binding in Nanoparticle Sandwich Immunoassays. Bioconjug. Chem..

[57] Paek S.-H., Lee S.-H., Cho J.-H., Kim Y.-S. (2000). Development of Rapid One-Step Immunochromatographic Assay. Methods.

[58] Zhang P., Liu X., Wang C., Zhao Y., Hua F., Li C., Yang R., Zhou L. (2014). Evaluation of Up-Converting Phosphor Technology-Based Lateral Flow Strips for Rapid Detection of Bacillus Anthracis Spore, Brucella Spp., and Yersinia Pestis. PLoS ONE.

[59] Latiano A., Tavano F., Panza A., Palmieri O., Niro G.A., Andriulli N., Latiano T., Corritore G., Gioffreda D., Gentile A. (2021). False-Positive Results of SARS-CoV-2 IgM/IgG Antibody Tests in Sera Stored before the 2020 Pandemic in Italy. Int. J. Infect. Dis..

[60] Wang Q., Du Q., Guo B., Mu D., Lu X., Ma Q., Guo Y., Fang L., Zhang B., Zhang G. (2020). A Method To Prevent SARS-CoV-2 IgM False Positives in Gold Immunochromatography and Enzyme-Linked Immunosorbent Assays. J. Clin. Microbiol..

[61] Barreira G.A., dos Santos E.H., Pereira M.F.B., Rodrigues K.A., Rocha M.C., Kanunfre K.A., Marques H.H.d.S., Okay T.S., Eisencraft A.P., Rossi Junior A. (2022). Technical Performance of a Lateral Flow Immunoassay for Detection of Anti-SARS-CoV-2 IgG in the Outpatient Follow-up of Non-Severe Cases and at Different Times after Vaccination: Comparison with Enzyme and Chemiluminescent Immunoassays. Rev. Inst. Med. Trop. S. Paulo.

[62] Hedman K., Seppälä I. (1988). Recent Rubella Virus Infection Indicated by a Low Avidity of Specific IgG. J. Clin. Immunol..

[63] Hedman K., Lappalainen M., Söderlund M., Hedman L. (1993). Avidity of IgG in Serodiagnosis of Infectious Diseases. Rev. Res. Med. Microbiol..

[64] Correa V.A., Rodrigues T.S., Portilho A.I., Trzewikoswki de Lima G., De Gaspari E. (2021). Modified ELISA for Antibody Avidity Evaluation: The Need for Standardization. Biomed. J..

[65] Wang Q., Lei Y., Lu X., Wang G., Du Q., Guo X., Xing Y., Zhang G., Wang D. (2019). Urea-Mediated Dissociation Alleviate the False-Positive Treponema Pallidum-Specific Antibodies Detected by ELISA. PLoS ONE.

[66] Yang J.Y., Burkert O., Mizaikoff B., Smiatek J. (2024). Impact of Urea on Monoclonal Antibodies: Multiple Destabilization and Aggregation Effects for Therapeutic Immunoglobulin G Proteins. ACS Omega.

[67] Rohrman B.A., Leautaud V., Molyneux E., Richards-Kortum R.R. (2012). A Lateral Flow Assay for Quantitative Detection of Amplified HIV-1 RNA. PLoS ONE.

[68] Altman D.G., Bland J.M. (1994). Statistics Notes: Diagnostic Tests 1: Sensitivity and Specificity. BMJ.

[69] Altman D.G., Bland J.M. (1994). Statistics Notes: Diagnostic Tests 2: Predictive Values. BMJ.

[70] Landis J.R., Koch G.G. (1977). The Measurement of Observer Agreement for Categorical Data. Biometrics.

[71] Sheoran A.S., Holmes M.A. (1996). Separation of Equine IgG Subclasses (IgGa, IgGb and IgG(T)) Using Their Differential Binding Characteristics for Staphylococcal Protein A and Streptococcal Protein G. Vet. Immunol. Immunopathol..

[72] Antigen-Detection in the Diagnosis of SARS-CoV-2 Infection. https://www.who.int/publications/i/item/antigen-detection-in-the-diagnosis-of-sars-cov-2infection-using-rapid-immunoassays.

[73] Rondaan C., Gard L., Niesters H.G.M., van Leer-Buter C., Zhou X. (2023). COVID or No COVID: Interpreting Inconclusive SARS-CoV-2 qPCR Results in Different Populations and Platforms. J. Clin. Virol. Plus.

[74] Sayed R.H., Abousenna M.S., Elsaady S.A., Soliman R., Saad M.A. (2022). Development of Lateral Flow Immunochromatographic Test for Rapid Detection of SARS-CoV-2 Virus Antigens in Clinical Specimens. Nanomaterials.

[75] D Souza S., Obeid W., Hernandez J., Hu D., Wen Y., Moledina D.G., Albert A., Gregg A., Wheeler A., Philbrook H.T. (2024). The Development of Lateral Flow Devices for Urinary Biomarkers to Assess Kidney Health. Sci. Rep..

[76] He Z.-X., Shi L.-C., Ran X.-Y., Li W., Wang X.-L., Wang F.-K. (2016). Development of a Lateral Flow Immunoassay for the Rapid Diagnosis of Invasive Candidiasis. Front. Microbiol..

[77] Chan C.P.Y., Sum K.W., Cheung K.Y., Glatz J.F.C., Sanderson J.E., Hempel A., Lehmann M., Renneberg I., Renneberg R. (2003). Development of a Quantitative Lateral-Flow Assay for Rapid Detection of Fatty Acid-Binding Protein. J. Immunol. Methods.

[78] Federation of Animal Sciences Societies (2010). Guide for the Care and Use of Agricultural Animals in Research and Teaching.

[79] Expert Committee on Biological Standardization (2016). WHO Guidelines for the Production, Control and Regulation of Snake Antivenom Immunoglobulins.

[80] Committee for Proprietary Medicinal Products (2022). Note for Guidance on Production and Quality Control of Animal Immunoglobulins and Immunosera for Human Use.

