# Supplementary material for: Overcoming Limited Access to Virus Infection Rapid Testing: Development of a Lateral Flow Test for SARS-CoV-2 with Locally Available Resources
Source: Biosensors (Basel). 2024 Aug 27;14(9):416. doi: 10.3390/bios14090416 (PMC11431090; doi:10.3390/bios14090416)
Supplement: Supplementary file 1 [file biosensors-14-00416-s001.zip › biosensors-3146400-supplementary.pdf]

biosensors

Supplementary materials

Overcoming Limited Access to Virus Infection Rapid Testing: Development of a Lateral Flow Test for SARS-Cov-2 with Locally Available Resources

Estefanía S. Peri Ibáñez [1,2], Agostina Mazzeo [2], Carolina Silva [2,3], Maria Juliana Juncos [2], Guadalupe S. Costa Navarro [2], Horacio M. Pallarés [2], Virginia J. Wolos [4], Gabriel L. Fiszman [4], Silvia L. Mundo [5], Julio J. Caramelo [2], Marcelo J. Yanovsky [2], Matías Fingermann [6], Alejandro A. Castello [1,7,8], Andrea V. Gamarnik [2], Ana S. Peinetti [3,*] and Daiana A. Capdevila [2,*]

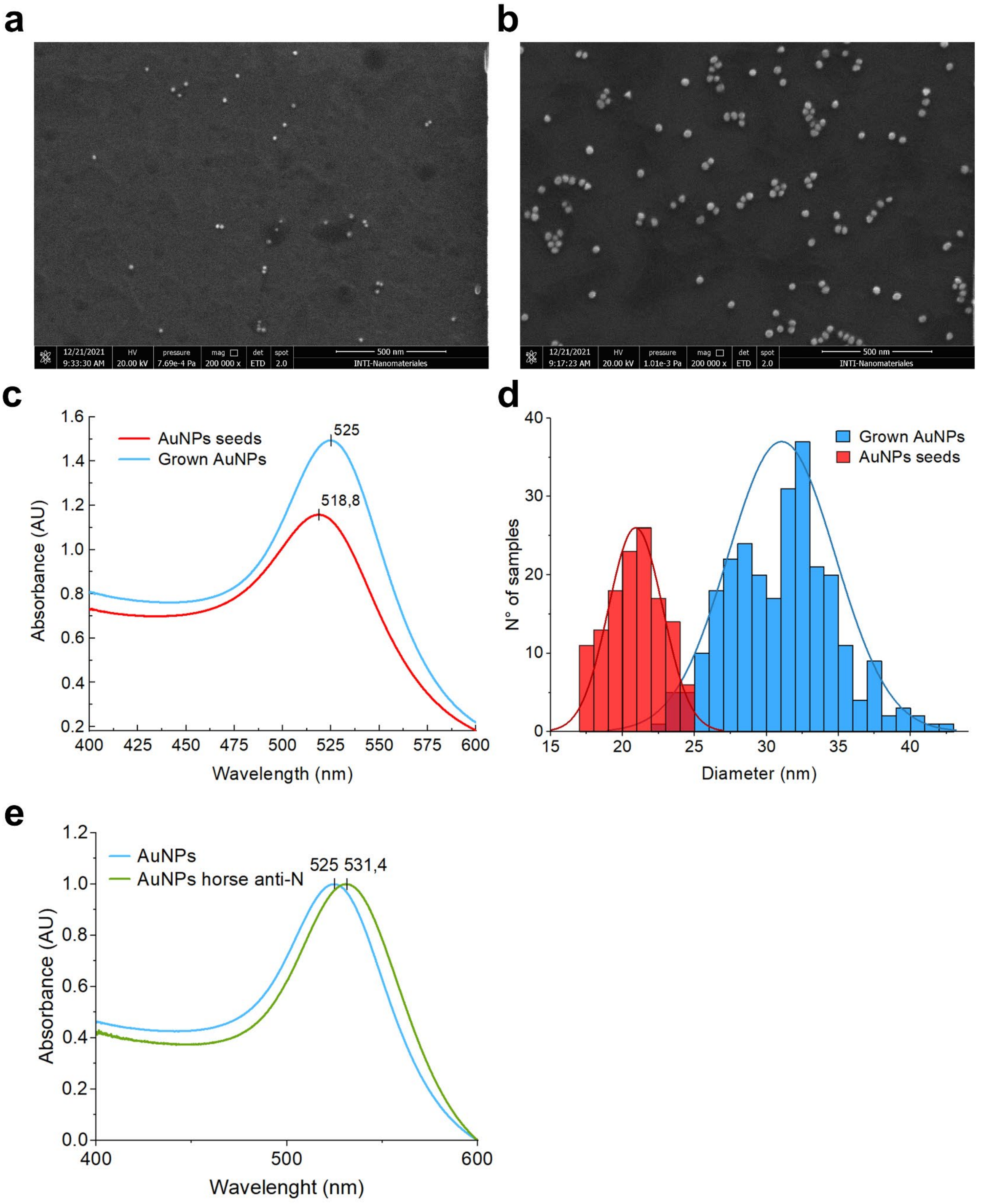

Figure S1. (a) Image taken by SEM of a 1:10 dilution of the AuNP seeds, where the spherical shape of the seeds is observed; (b) Image taken by SEM of an undiluted solution of grown AuNPs, where the shape is observed to be a little less spherical compared to the seeds.; (c) Spectra obtained by UV-vis measurement. The red curve corresponds to the 20 nm AuNPs, whose maximum absorbance peak is 519 nm and corresponds to 1.16 absorbance units. The red curve corresponds to the 30 nm AuNPs for which a change in the spectrum is observed towards longer wavelengths, as corresponds to larger sizes of the AuNPs. In this case the maximum absorbance peak is 525 nm and corresponds to 1.3 absorbance units; (d) Histograms with the frequency of AuNP seed sizes (red), and grown AuNP sizes (blue), showing a normal distribution. The average size is 21 nm, with a standard deviation of 2 nm for the seeds and 31 nm, with a 3 nm standard deviation for the grown AuNPs. Comparing both histograms, it can be seen the effective growth of the AuNPs; (e) Spectra obtained by UV-vis measurement. The blue curve corresponds to 31 nm AuNPs, whose maximum absorbance peak is 525 nm. The green curve corresponds to AuNPs horse anti-N conjugates and shows a shift of the absorbance maximum of 531,4 nm. The increase in absorbance values can correlate with the conjugation process producing larger sizes of the AuNPs.

Scanning electron microscopy experiments were performed using a Thermo Fisher Quanta 250 microscope, available at Instituto Nacional de Tecnología Industrial (INTI), Buenos Aires, Argentina. The resulting images were analyzed using ImageJ software.

<table>
<thead>
<tr><th>Antibody</th><th>Optimized pH</th><th>Optimized concentration</th></tr>
</thead>
<tbody>
<tr><td>pAb horse anti-N
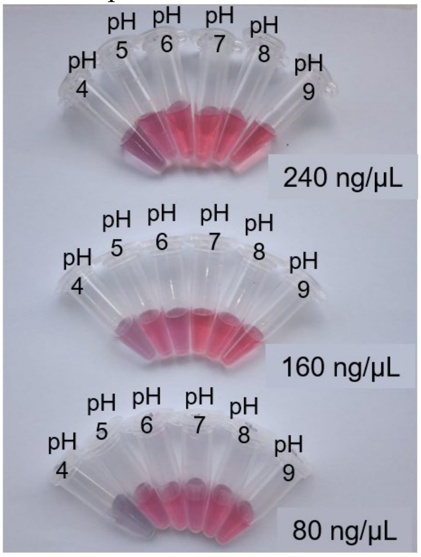
</td><td>7</td><td>80 ng/µL</td></tr>
<tr><td>mAb mouse anti-N
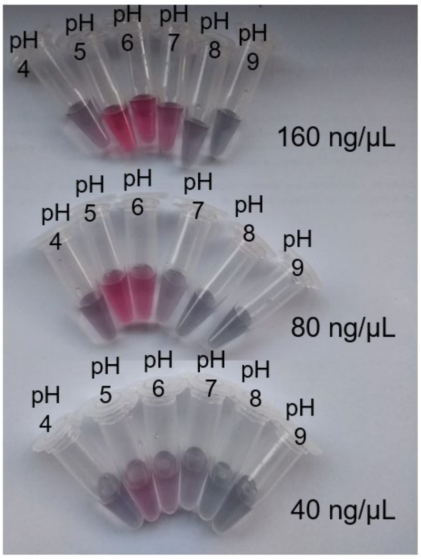
</td><td>6</td><td>160 ng/µL</td></tr>
<tr><td>mAb rabbit anti-SARS-CoV-2 NP, clon NJ1, Creative Diagnostics
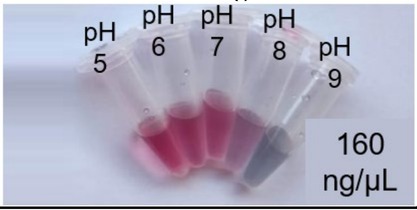
</td><td>6</td><td>160 ng/µL</td></tr>
</tbody>
</table>

Table S1. Conjugation parameters tested and selected for each antibody.

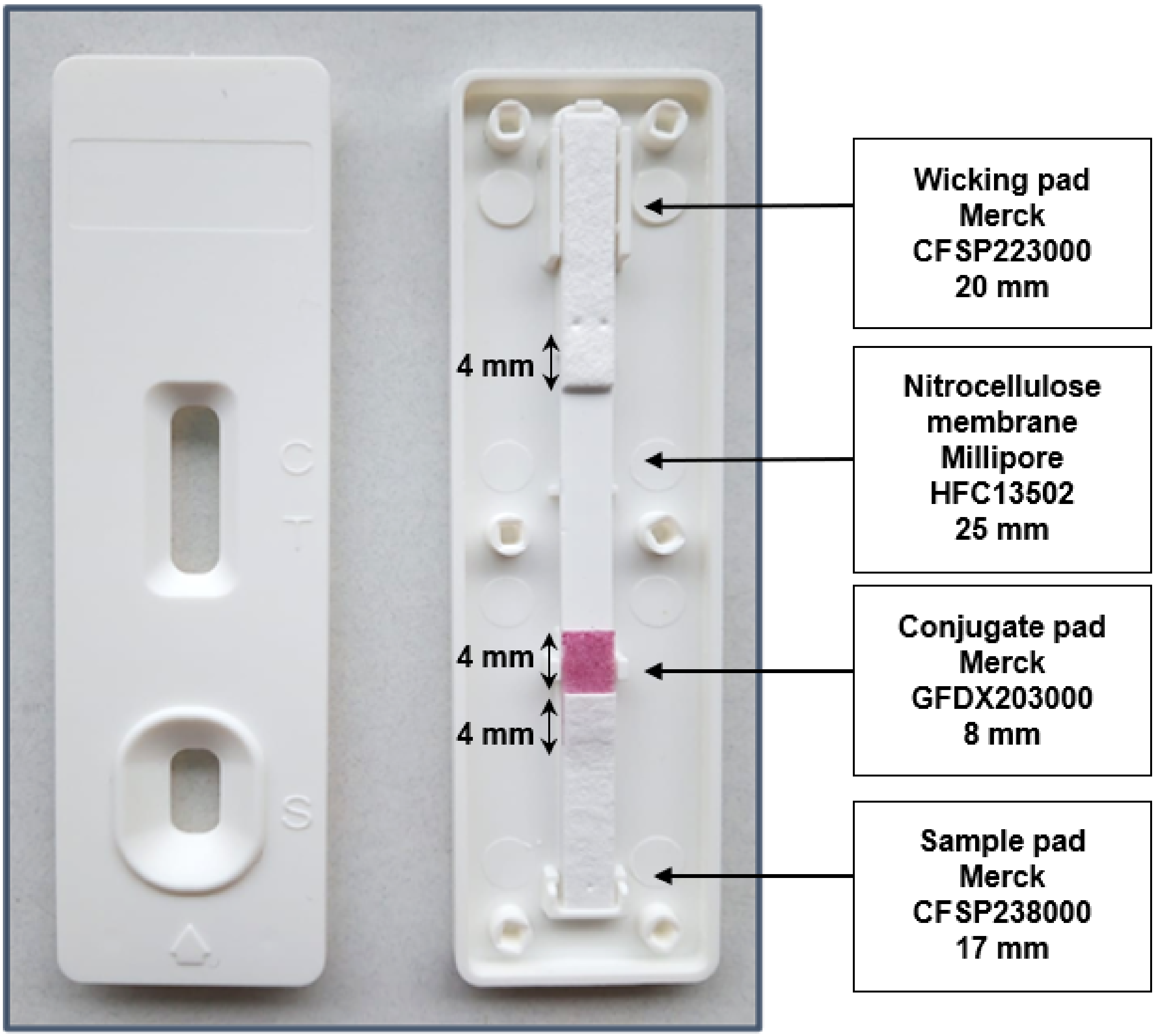

Figure S2. Full strip final conformation with 60 mm x 3.7 mm dimensions placed inside the commercial LFT cassette (Zhuhai Ideal Plastic Product Co., Ltd., Zhuhai City, Guangdong, China. A 25 mm x 300 mm Millipore HFC13502 nitrocellulose membrane was assembled on a 60 mm x 300 mm backing card from DCN Diagnostics, on the corresponding section marked for this purpose. Afterward, the membrane was dispensed, blocked, dried, and stored. Merck CFSP223000 cellulose wicking pad was cut into 20 mm x 300 mm pieces and stored. Merck CFSP238000 cellulose sample pad was cut into 17 mm x 300 mm pieces and pre-treated with sample buffer, dried, and then stored. Merck GFDX203000 glass fiber conjugate pad was cut into 8 mm x 300 mm pieces, dispensed, dried, and stored. All pads were assembled on the corresponding section marked on the backing card overlapping 4 mm between them to ensure a continuous flow through the strip, using a BioDot LM5000™ manual lamination system and then cut into 3.7 mm strips using a BioDot CM5000™ guillotine cutting module.

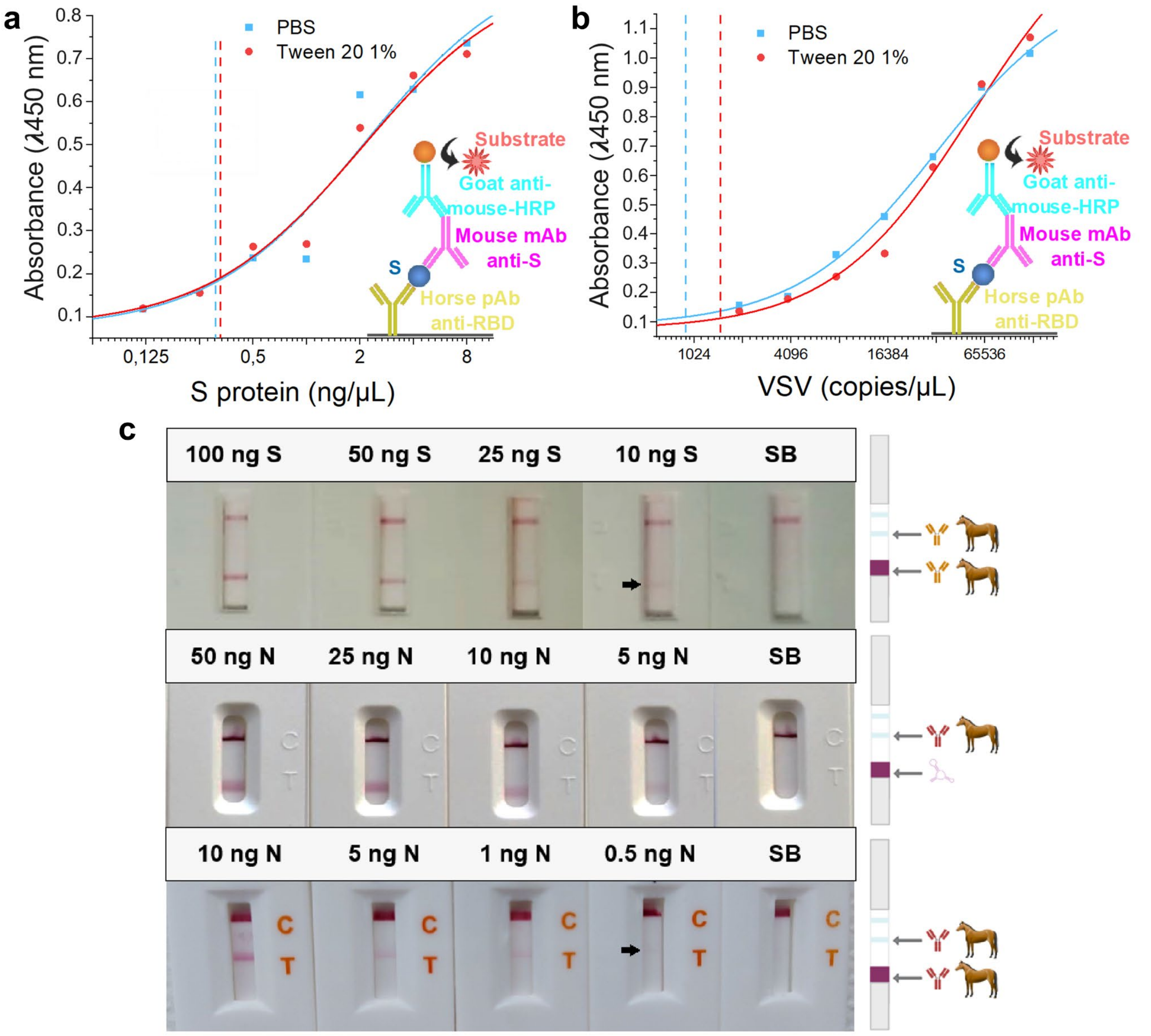

Figure S3. Dose-response relationship curve obtained from ELISA sandwich assays using plates sensitized with horse polyclonal anti-RBD pAb (at 50 ng per well, at 4ºC ON). In the second step of the assay, the plates were incubated with (a) the ectodomain of the SARS-CoV-2 spike protein at 37ºC for 1 hour or (b) VSV pseudotyped with SARS-CoV-2 spike. The assays were carried out in PBS solution (blue) and PBS-1% Tween 20 (red). Detection was first performed with mouse monoclonal anti-spike as the primary antibody (at 65 µg/mL, at 37ºC for 1 hour) and then with commercial HRP-conjugated Abs (goat anti-mouse-HRP, R&D Systems, Inc., United States), according to the manufacturer's instructions, measuring the optical density of the samples at 450 nm. The detection limit (indicated with a dotted line) was obtained from the upper limit of the IC95 of the fit to a rectangular hyperbola (solid line) evaluated at (a) 0.06 ng/100 µL and (b) 800 copies of VSV/100 µL; (c) Full strip assay results comparing S and N protein detection using sample buffer spiked with these proteins. The S protein detection strips (top panel) were dispensed with 1 µg of horse pAbs anti-RBD and a conjugate using the same Ab. In this instance, a faint line was visible up to 10 ng of S protein. For N protein detection, both strategies used implied strips dispensed with 1 µg of horse pAbs anti-N, but two different conjugates, aptamer anti-N conjugate (middle panel) and horse pAbs anti-N conjugate (bottom panel). In the case of aptamer-based detection, a slightly faint line was visible up to 5 ng of N protein. On the other hand, pAbs-based detection showed a lower limit of detection with a faint line visible up to 0.5 ng of N protein.

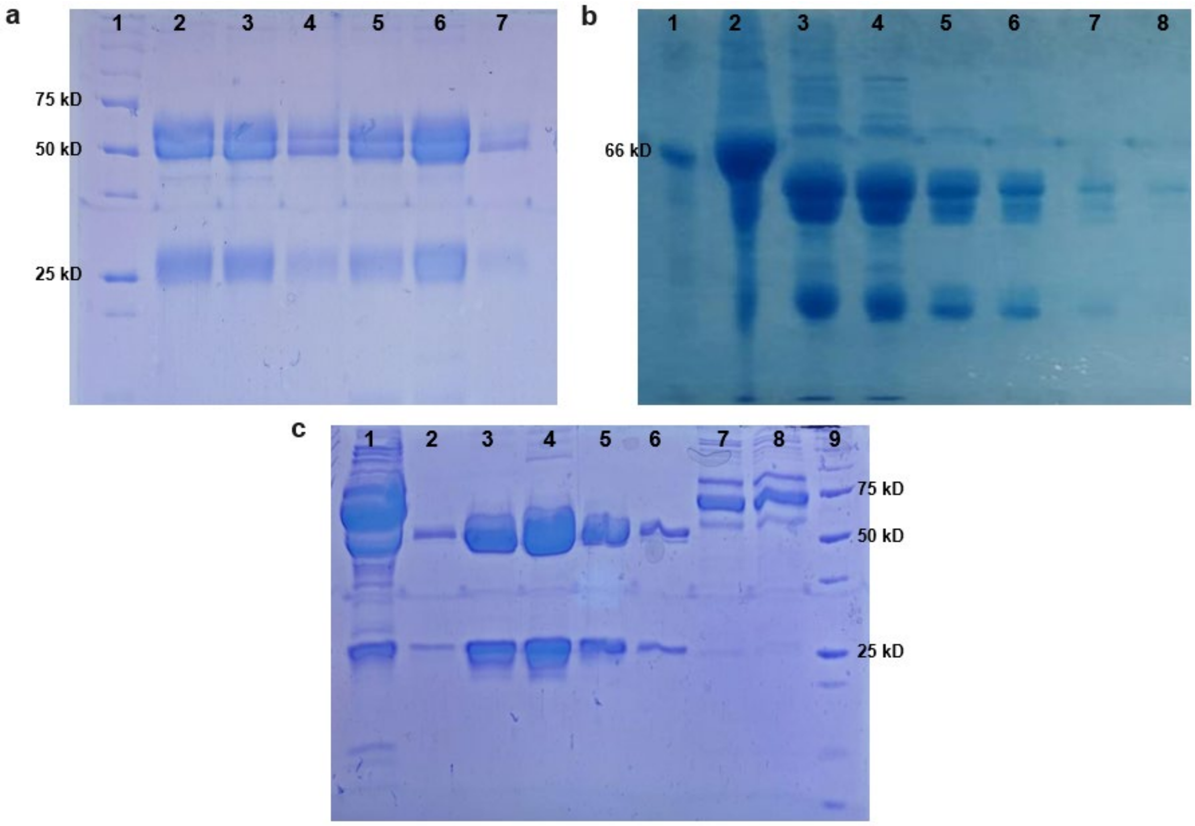

Figure S4. (a) SDS-PAGE of horse pAbs purification process. 1) Molecular weight marker Precision plus protein standard (BioRad); 2) Supernatant after caprylic acid precipitation; 3) N protein affinity column flow-through; 4) PBS buffer wash; 5) Purified fraction (Tris-Gly pH 7); 6) Purified fractions pool; 7) Glycine buffer wash; (b) SDS-PAGE of llama pAbs purification process. 1) BSA pattern; 2) Unpurified llama sera; 3) Supernatant after caprylic acid precipitation; 4) N protein affinity column flow-through; 5-8) Purified fractions (Tris-Gly pH 7); (c) SDS-PAGE of mouse mAbs protein G purification process. 1) Mouse ascitic fluid (1:10 dilution); 2-6) Purified fractions (Tris-Gly pH 7); 7-8) Protein G column flow-through; 9) Molecular weight marker Precision plus protein standard (BioRad).

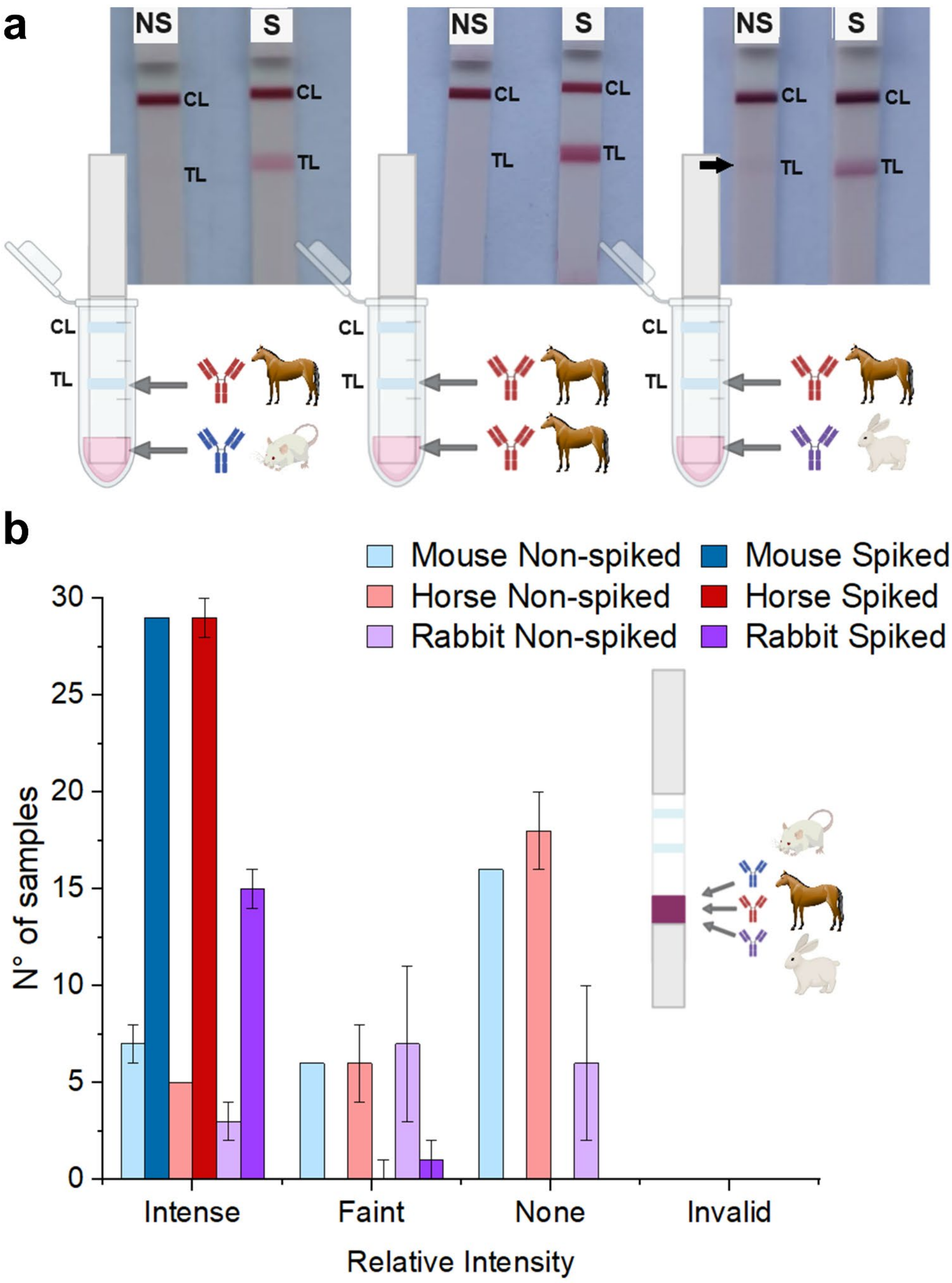

Figure S5. (a) Half-strip assay results using sample buffer with (S) and without (NS) inactivated virus (Ct=26) with three different anti-N conjugates: horse pAb, mouse mAb, and commercial rabbit mAb anti-SARS-CoV-2 NP, clon NJ1, Creative Diagnostics. In all three cases, an intense signal can be seen on the TL, stronger in the case of horse conjugate. A faint non-specific signal can be seen on the TL on the rabbit conjugate's NS assay, indicating a possible NSB problem with this particular conjugate. In all cases, the strips were dispensed with 1 μg horse anti-N per strip on the test line on a Sartorius UniSart CN95 membrane; (b) Histograms showing the relative intensity (qualitatively) results for full-strip assays using spiked (S, inactivated virus particles) or non-spiked (NS, deionized water) RT-qPCR negative nasopharyngeal swab samples with three different anti-N conjugates: horse pAb, mouse mAb, and commercial rabbit mAb anti-SARS-CoV-2 NP, clon NJ1, Creative Diagnostics. The commercial rabbit mAb conjugate had the lowest rate of false positives for non-spike samples but also showed a decrease in signal intensity for spiked samples. Both mouse mAb and horse pAb conjugates showed a high rate of intense signals for spiked samples, with the horse pAb conjugate exhibiting a higher decrease in signals for non-spiked samples. After considering a balance between sensitivity and specificity, the horse pAb conjugate was chosen. In all cases, the strips were dispensed with 1 μg horse anti-N per strip on the test line on a Sartorius UniSart CN95 membrane.

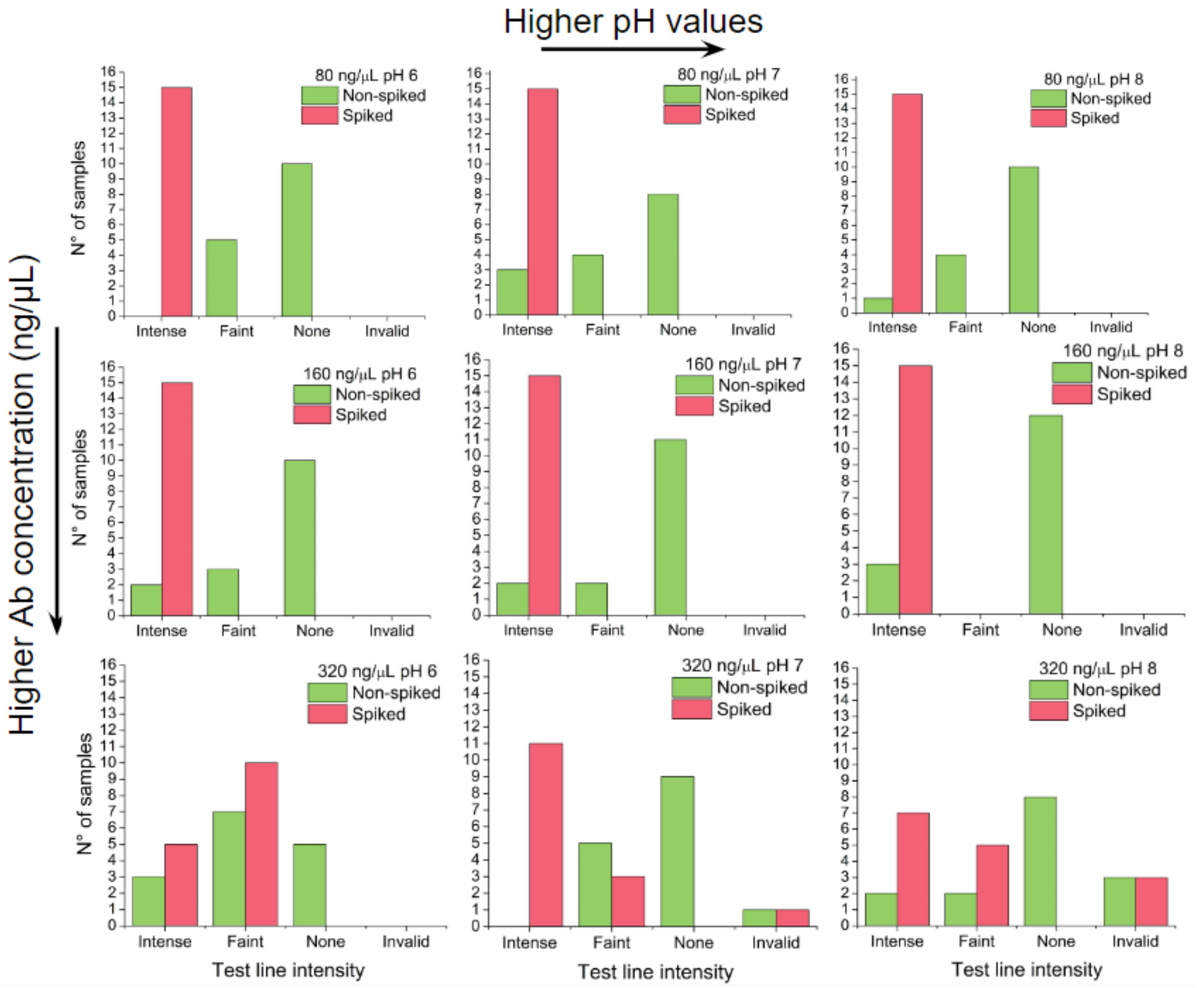

Figure S6. Histograms showing the performance of full-strip assays using horse conjugates under various conditions, as indicated by the relative intensity of the test line. Antibody concentrations of 80 ng/μL, 160 ng/μL, and 320 ng/μL, at pH levels of 6, 7, and 8, are presented as conjugation conditions. Increasing antibody concentration leads to a decrease in the overall performance of the assay, with occurrences of invalid tests and/or false positives. Additionally, higher concentrations result in a fainter test line colorimetric intensity. Conversely, in stability tests, higher antibody concentrations lead to more stable conjugates across a wider pH range. However, a pH value of 8 for conjugation appears to have a negative impact on test performance. In all cases, the strips were dispensed with 1 μg horse anti-N per strip on the test line on a Sartorius UniSart CN95 membrane.

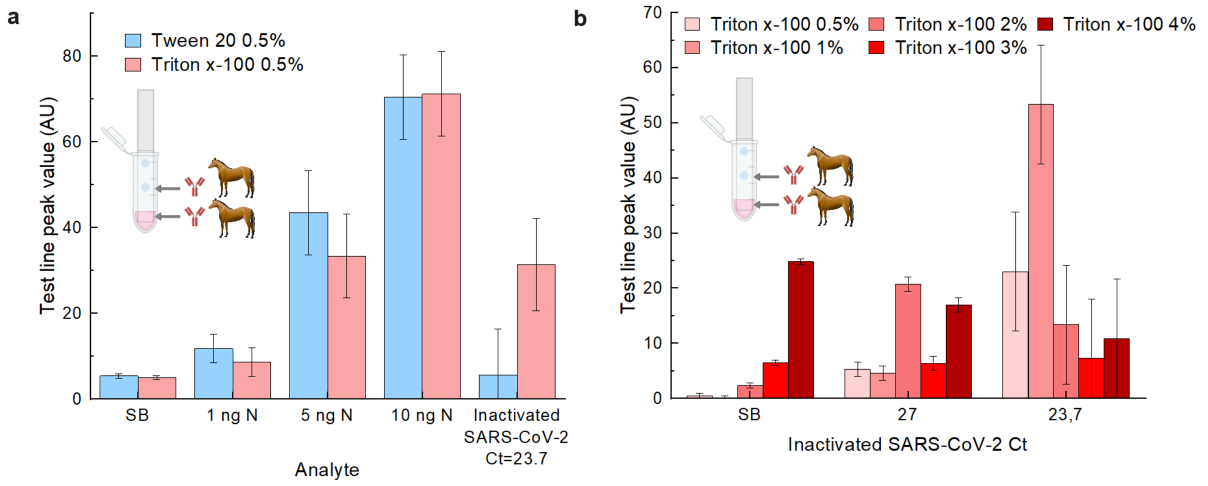

Figure S7. (a) Histograms showing half-strip assay results using two different detergents, Tween 20 and Triton x-100 in sample buffer comparison. Sample buffer was tested by adding various concentrations of N protein and

inactivated SARS-CoV-2 dilutions from a Ct=23 stock, or without any addition. N protein spiked buffer showed a higher signal for the assays with Tween 20. This could be attributed to the fact that Triton x-100 may affect the stability of the recombinant protein. Both signals are comparable when an excess of N protein is added. On the other hand, in virus-spiked spiked buffer, a considerably higher signal is observed when Triton x-100 is used, which may indicate that this detergent functions better for exposing the antigen; (b) Histograms showing half-strip assay results obtained using increasing concentrations of Triton x-100 in the sample buffer comparison. Sample buffer was testing adding different dilutions of inactivated SARS-CoV-2 or without any addition. The results with virus dilutions show good results with concentrations between 0.5 and 2%. However, using only sample buffer, detergent concentrations from 2 to 4% showed an increment in the non-specific signal. Considering this, we selected a 1% concentration of Triton x-100. In all cases, the strips were dispensed by hand with 1 μg horse anti-N per strip on the test line on a Sartorius UniSart CN95 membrane and using AuNPs-horse pAb conjugate. Test line peak values were determined by image analysis with ImageJ software.

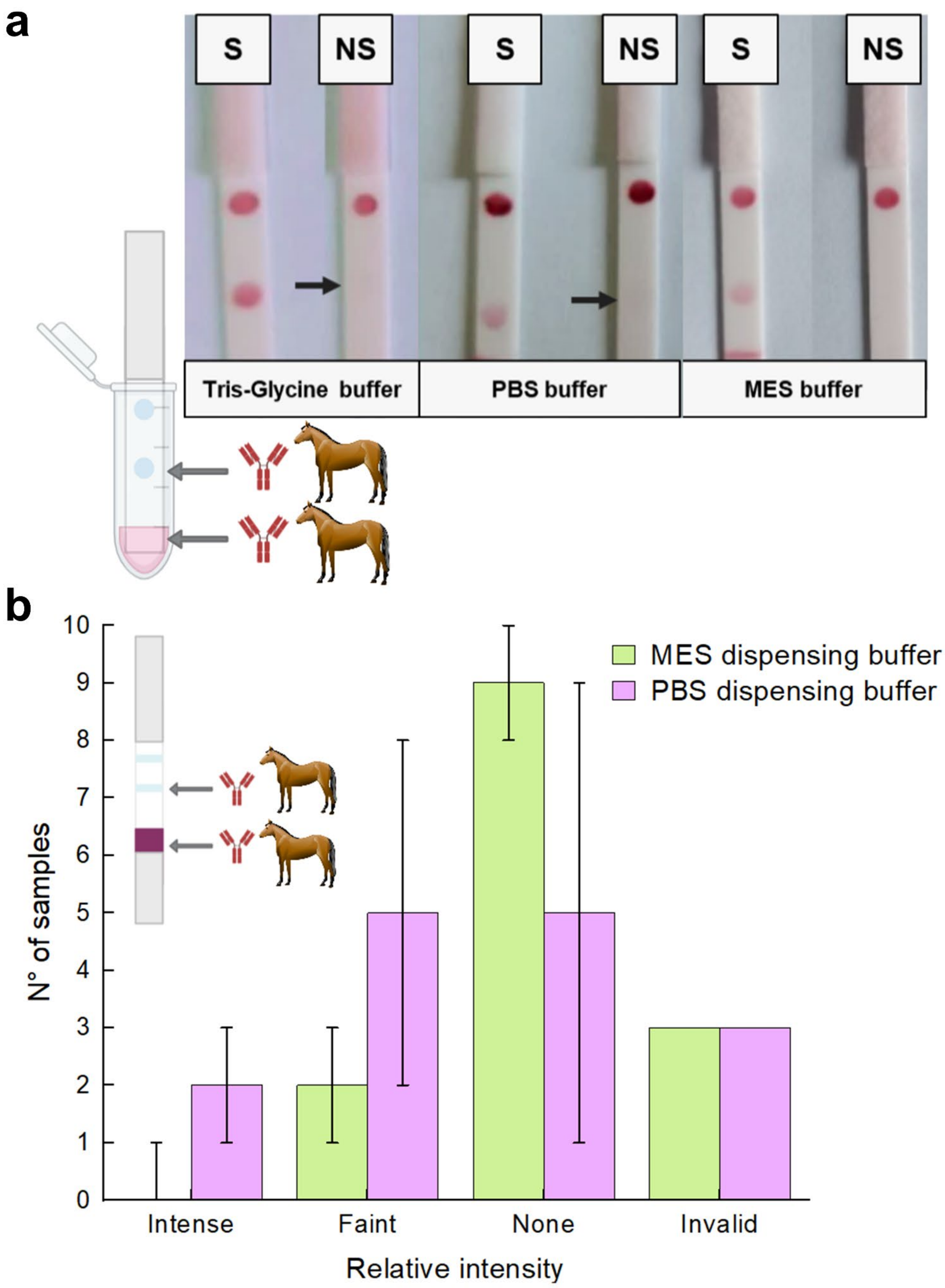

Figure S8. (a) Half-strip assay results for horse pAbs dispensed in different buffers, Tris-Glycine buffer pH 7, obtained from post affinity purification column (elution in glycine buffer 0.1 M pH 2.8 and collected in 10% Tris buffer 1 M pH 8), PBS pH 7.4, and MES 25 mM pH 6.5. Sample buffer (PBS + Triton x-100 1%) was tested with (Spiked (S)) or without (Non-spiked (NS) 10 ng of N protein. N protein spiked buffer showed a strong signal in the three cases. However, in non-spiked cases, a faint signal can be seen for both Tris-Glycine and PBS buffers, but no signal can be seen with MES buffer. For all cases, strips were dispensed by hand with 1 µg horse anti-N per strip on the test line on a Sartorius UniSart CN95 membrane and using AuNPs-horse pAb conjugate; (b) Histograms showing the relative intensity (qualitatively) results for full strip assays with the test line dispensed in PBS or MES buffer using non-spiked RT-qPCR negative nasopharyngeal samples. MES-dispensed tests showed a decrement in non-specific signals, showing only none or faint test signals. On the contrary, PBS-dispensed tests showed for some

samples intense test signals. For both cases, the same amount of invalid tests was observed. In all cases, the strips' test lines were dispensed with 1 µg horse anti-N per strip on a Sartorius UniSart CN95 membrane.

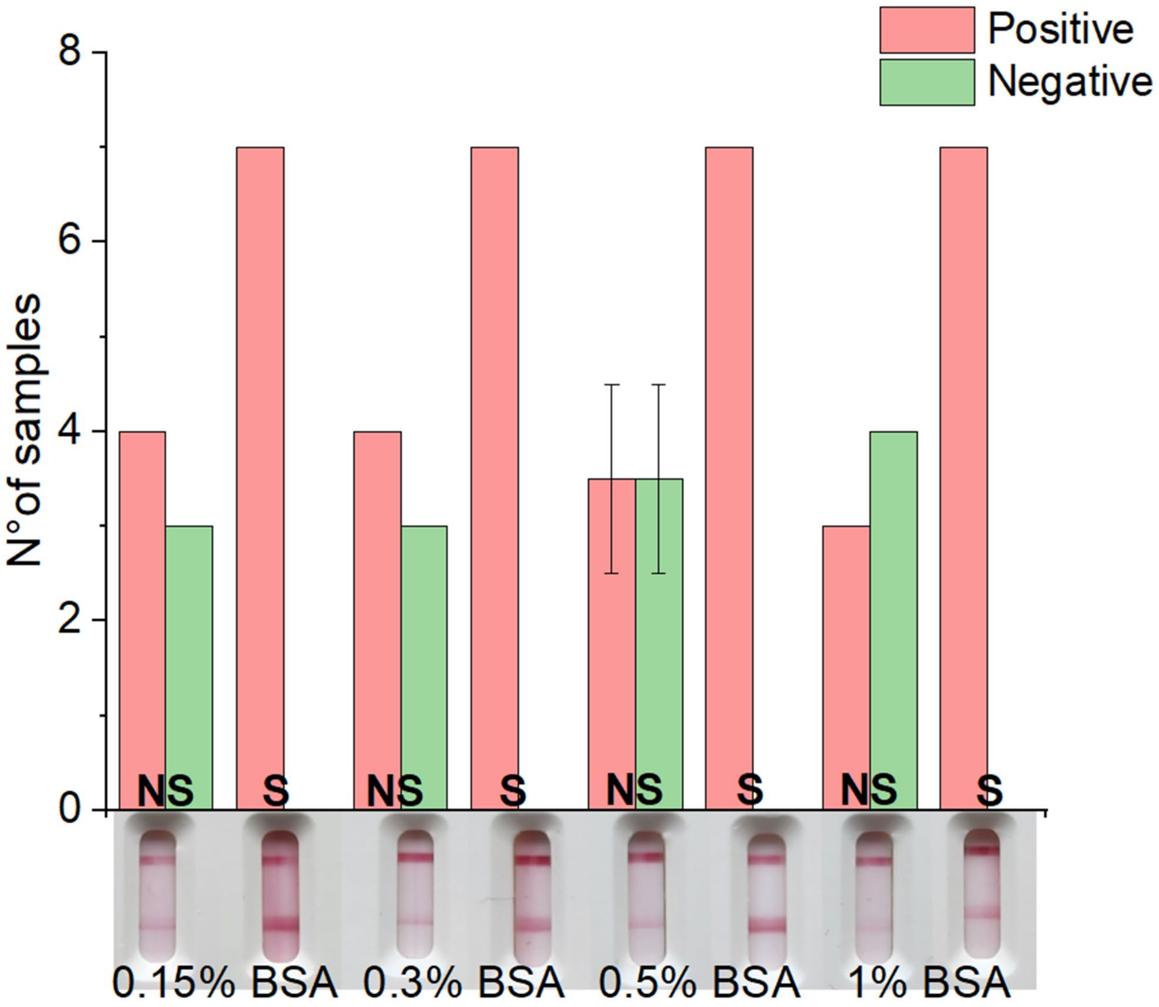

Figure S9. Full strip assays using spiked (S, inactivated virus, Ct=26) and non-spiked (NS, deionized water) RT-qPCR nasopharyngeal swab samples ran in UniSart CN95 membranes previously blocked with increasing amounts of BSA. In all cases, the strips' test lines were dispensed with 2 µg horse anti-N per strip.

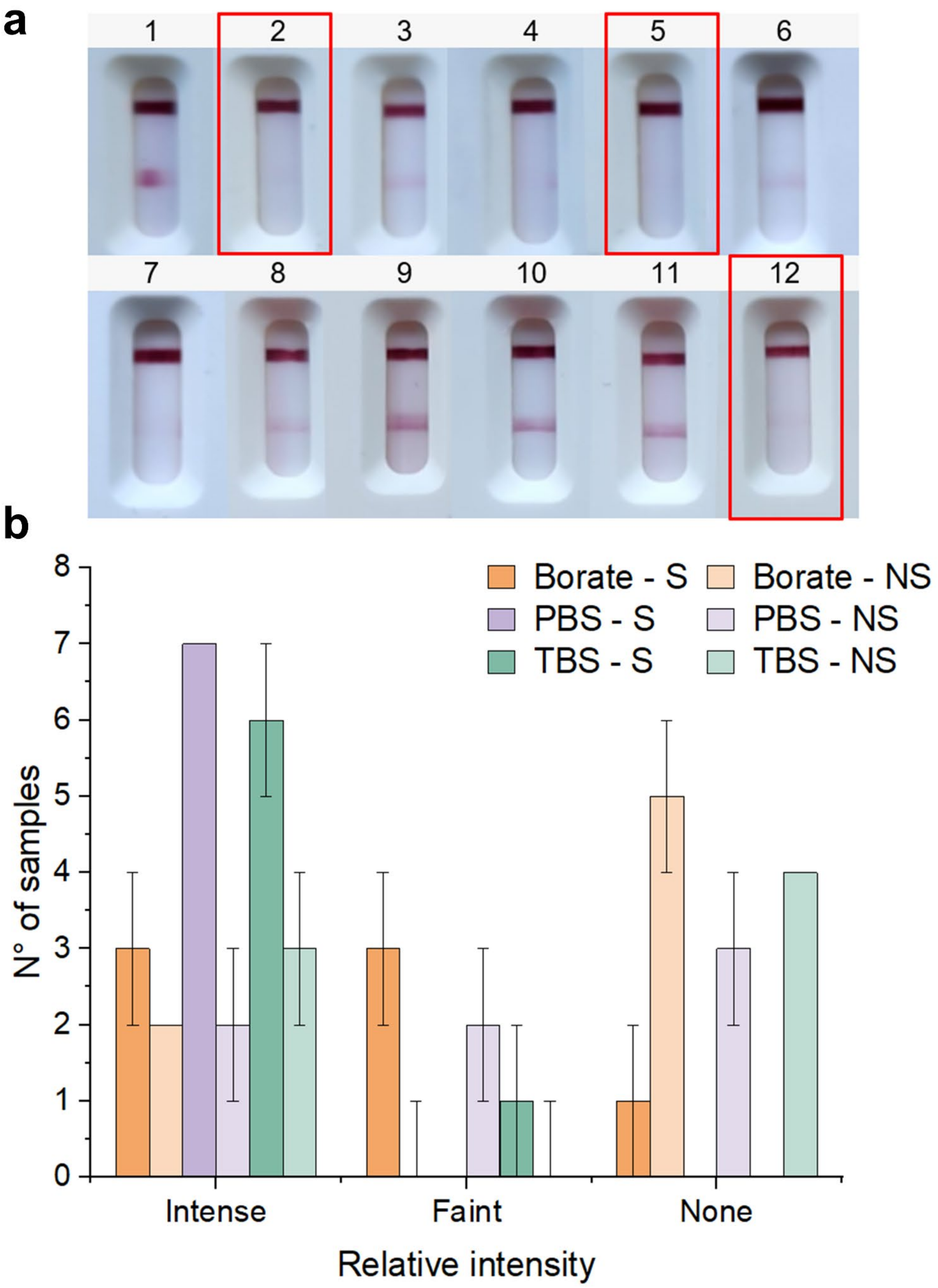

Figure S10. (a) Full-strip assay results using sample buffers with different molarities, pH ranges, and BSA and detergent concentrations tested with non-spiked RT-qPCR negative nasopharyngeal swab sample that displayed a strong non-specific signal in the absence of viral particles with the optimized test strips: 1) Sodium borate 10 mM, pH 10 + NaCl 1,37 M + BSA 0,02% + Tween 20 0,5%; 2) Sodium borate 10 mM, pH 10 + BSA 0,02% + Tween 20 0,5%; 3) Sodium carbonate 10 mM, pH 11 + NaCl 1,37 M + BSA 0,02% + Tween 20 0,5%; 4) Sodium carbonate 10 mM, pH 10 + BSA 0,02% + Tween 20 0,5%; 5) TBS pH 7.5 + BSA 0,02% + Tween 20 0,5%; 6) Tricine 100 mM, pH 7.5 + NaCl 100 mM + Tween 20 1%; 7) PBS pH 7.2 + BSA 0,2% + Tween 20 0,5%; 8) Sodium borate 100 mM, pH 8 + BSA 0,02% + Tween 20 0,5%; 9) Sodium borate 100 mM, pH 8 + BSA 0,2% + Tween 20 1%; 10) TBS pH 8.2 + BSA 0,2% + Tween 20 1%; 11) Tricine 100 mM, pH 8.4 + Tween 20 1%; 12) PBS pH 7.2 + Tritón 1%. Strips were dispensed with 2 µg horse anti-N in MES 25 mM pH 6.5 per strip on the test line on a Millipore HFC13502 membrane and using AuNPs-horse pAb conjugate. A range of test line intensities can be observed in all cases, with the lowest intensity observed for buffers 2, 5, and 12; (b) Histograms showing the relative intensity (qualitatively) results for full-strip assays using spiked (S, inactivated virus particles) or non-spiked (NS, deionized water) RT-qPCR negative nasopharyngeal swab samples with the three previous selected sample buffers: Sodium borate 10 mM, pH 10 + BSA 0,02% + Tween 20 0,5%, TBS pH 7.5 + BSA 0,02% + Tween 20 0,5% and, PBS pH 7.2 + Tritón 1%. While the borate-based buffer had the lowest rate of false positives for non-spike samples, it was also the only buffer that

resulted in no signal for a proportion of spiked samples. The TBS-based buffer displayed a high rate of spiked samples with intense signals, but in some cases, faint signals were observed. Additionally, it had the highest rate of non-spike samples with intense signals. On the other hand, the PBS-based sample buffer consistently showed a strong signal in the TL for all spiked samples, despite also producing intense and faint signals for non-spiked samples. Considering a balance between sensitivity and specificity, the PBS-based buffer was selected. In all cases, strips were dispensed with 2 μg horse anti-N in MES 25 mM pH 6.5 per strip on the TL on a Millipore HFC13502 membrane and using AuNPs-horse pAb conjugate.

Discussion S1: Next, we evaluated the sample buffer composition, another critical component for the overall LFT performance to modulate and reduce NSB [48,49]. Mucus samples are usually found in a pH range that can vary between 5.5 and 8.3 [3], for this reason, the buffers used for swab' extracting and running can be diverse. Various salts, surfactants, and macromolecules are usually included in the sample buffer and need to be optimized for each assay mostly because an LFT that accurately detects the target antigen in solution will not perform similarly when evaluating samples containing viral particles, free viral components, and antigen-bearing cell debris to which the antigen must be properly exposed first. As previously described in section 3.2., to this point, we used a sample buffer composed of PBS, pH 7.4 containing 1% of Triton x-100, to reduce any hydrophobic interactions and to decrease the surface tension of the sample buffer, as it allowed a broader dynamic range and performed better at high concentrations of viral particles. Regarding salts and macromolecules, we conducted tests on various buffer solutions with different molarity and pH ranges. It is known that pH, buffering capacity, and ionic strength are critical components to ensure proper test function and avoid the generation of false-positive results [35]. Moreover, ionic strength can eliminate NSB of immunological reactions at a proper concentration, but it can also damage specific binding at an unsuitably high concentration, as it tends to reduce antibody-antigen binding constants [58]. We also tested the effects of adding BSA. We evaluated non-spiked nasopharyngeal swab samples producing particularly high NSB with some of the most used buffers for LFT [3]: phosphate (pH range between 5.8 and 8), TRIS (pH range between 7.5 and 9), and borate (pH range between 8 and 10), in concentrations between 10 and 100 mM; as well as a sample buffer similar to the one used in the Abbott PANBIO™ COVID-19-Ag rapid test [35], as shown in Figure S10a. We selected the ones that exhibited the most significant decrease in NSB and tested them with a large number of spiked and non-spiked samples (Figure S10b). However, we still achieved the best results using the same buffer sample we had initially used, as in the other selected buffers, TL relative intensity for spiked samples also showed a decrease."

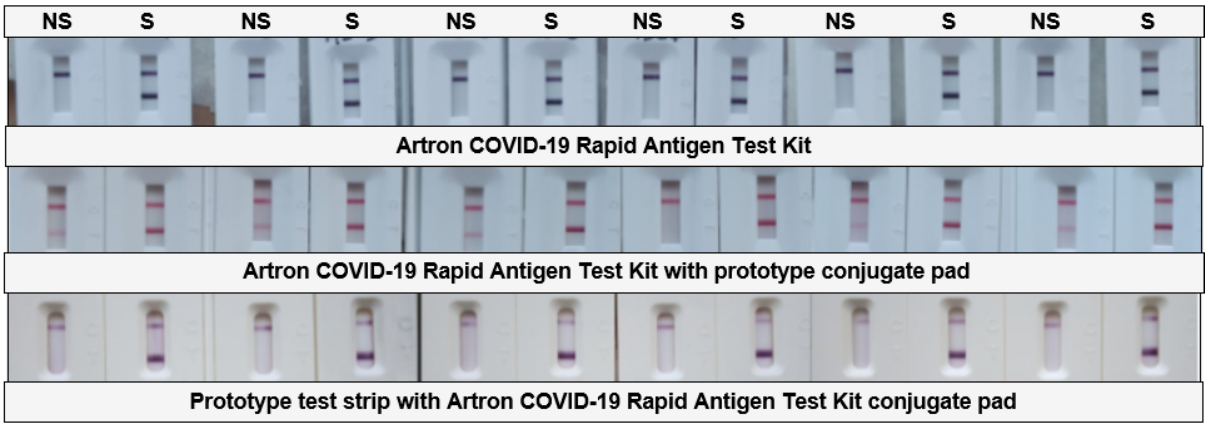

Figure S11. Full-strip assay results from 6 representative nasopharyngeal swabs out of 15 tested with commercial tests (Artron COVID-19 Rapid Antigen Test Kit), COVIDAR-Ag conjugate pad transplantation to a commercial test, and commercial conjugate pad transplantation to the COVIDAR-Ag test presented here (from top to bottom). The COVIDAR Ag test was obtained following the conditions in Table 1 and the commercial tests were performed as indicated by the manufacturer.

Prototype #	Conjugate OD	% of BSA blocking	% of PEG blocking	Pad pretreatment
**1**	15	2.1	-	-
**2**	15	0.5	0.2	-
**3**	15	0.5	-	-
**4**	10	0.5	0.2	-
**5**	10	0.5	-	-
**6**	10	2.1	-	-
**7**	10	2.1	-	Tween 20 0.5%

Table S2. Conjugate pad prototypes.

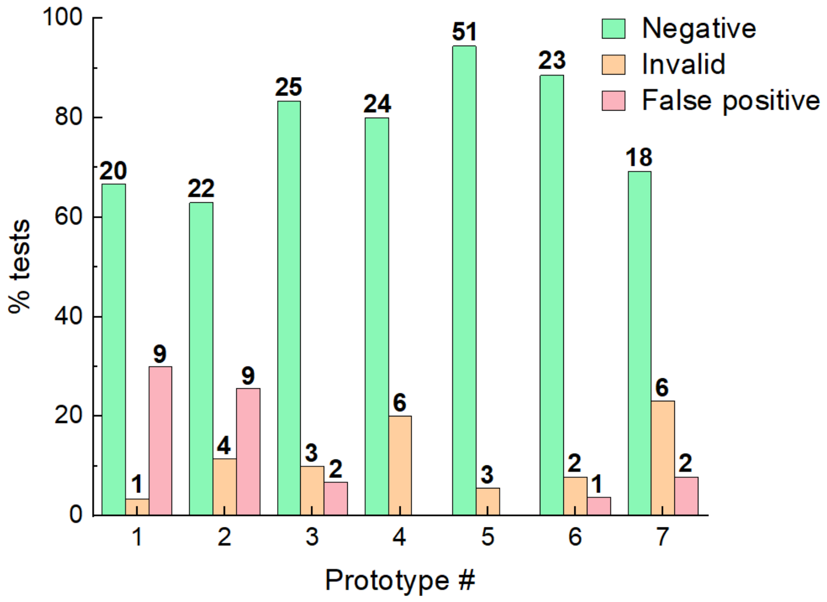

Figure S12. Histograms showing the relative amounts of negative, invalid, and false positive results obtained using the different conjugate pad prototypes are summarized in Table S2. The sample number is detailed on each bar. Only two prototypes, 4 and 5, showed no false positive results. Among these two, prototype 5 displayed a better performance, with only 5% of invalid tests in contrast with 20% of prototype 4%. In all cases, strips were dispensed with 2 µg horse anti-N in MES 25 mM pH 6.5 per strip on the TL on a Millipore HFC13502 membrane.
